# Oleoylethanolamide enhances β-adrenergic-mediated thermogenesis and white-to-brown adipocyte phenotype in epididymal white adipose tissue in rat

**DOI:** 10.1242/dmm.013110

**Published:** 2013-10-23

**Authors:** Juan Suárez, Patricia Rivera, Sergio Arrabal, Ana Crespillo, Antonia Serrano, Elena Baixeras, Francisco J. Pavón, Manuel Cifuentes, Rubén Nogueiras, Joan Ballesteros, Carlos Dieguez, Fernando Rodríguez de Fonseca

**Affiliations:** 1 Laboratorio de Medicina Regenerativa, Hospital Carlos Haya-IBIMA (Pabellón de Gobierno), Avenida, Carlos Haya 82, 29010 Málaga, Spain.; 2 Centro de Investigación Biomédica en Red Fisiopatología de la Obesidad y Nutrición (CIBERobn), Instituto de Salud Carlos III, Calle Sinesio Delgado 6, 28029 Madrid, Spain.; 3 Departamento de Biología Celular, Genética y Fisiología, Universidad de Málaga, Avenida, Louis Pasteur s/n, 29071 Málaga, Spain.; 4 Centro de Investigaciones Biomédicas en Red de Bioingeniería, Biomateriales y Nanomedicina (CIBER-BBN), Instituto de Salud Carlos III, Calle Sinesio Delgado 6, 28029 Madrid, Spain.; 5 Department of Physiology, School of Medicine-CIMUS, University of Santiago de Compostela-Instituto de Investigación Sanitaria, S. Francisco s/n, 15782 Santiago de Compostela, Spain.; 6 ViviaBiotech S.L., Severo Ochoa 35 (Edf. Bioanad), 29590 Campanillas, Spain.

**Keywords:** Peroxisome proliferator-activated receptor alpha, β3-adrenergic receptor, Thermogenesis, β-oxidation, Adipocyte

## Abstract

β-adrenergic receptor activation promotes brown adipose tissue (BAT) β-oxidation and thermogenesis by burning fatty acids during uncoupling respiration. Oleoylethanolamide (OEA) can inhibit feeding and stimulate lipolysis by activating peroxisome proliferator-activating receptor-α (PPARα) in white adipose tissue (WAT). Here we explore whether PPARα activation potentiates the effect of β3-adrenergic stimulation on energy balance mediated by the respective agonists OEA and CL316243. The effect of this pharmacological association on feeding, thermogenesis, β-oxidation, and lipid and cholesterol metabolism in epididymal (e)WAT was monitored. CL316243 (1 mg/kg) and OEA (5 mg/kg) co-administration over 6 days enhanced the reduction of both food intake and body weight gain, increased the energy expenditure and reduced the respiratory quotient (VCO_2_/VO_2_). This negative energy balance agreed with decreased fat mass and increased BAT weight and temperature, as well as with lowered plasma levels of triglycerides, cholesterol, nonessential fatty acids (NEFAs), and the adipokines leptin and TNF-α. Regarding eWAT, CL316243 and OEA treatment elevated levels of the thermogenic factors PPARα and UCP1, reduced p38-MAPK phosphorylation, and promoted brown-like features in the white adipocytes: the mitochondrial (*Cox4i1*, *Cox4i2*) and BAT (*Fgf21*, *Prdm16*) genes were overexpressed in eWAT. The enhancement of the fatty-acid β-oxidation factors *Cpt1b* and *Acox1* in eWAT was accompanied by an upregulation of *de novo* lipogenesis and reduced expression of the unsaturated-fatty-acid-synthesis enzyme gene, *Scd1*. We propose that the combination of β-adrenergic and PPARα receptor agonists promotes therapeutic adipocyte remodelling in eWAT, and therefore has a potential clinical utility in the treatment of obesity.

## INTRODUCTION

Despite initial expectations, data obtained over the last two decades have shown an almost complete failure of traditional pharmacological-based monotherapies for the treatment of obesity. To try to overcome this situation, the attention is now being focused on the development of polytherapies that, by exerting their effects in different biological process such as food intake and energy expenditure, will have a greater chance of success.

β3-adrenergic receptor, a G-protein-coupled receptor abundantly expressed in adipose tissues, is a potent target for anti-obesity and anti-diabetic drug therapy. Mice lacking the β-adrenergic receptors develop massive obesity (Bachman et al., 2002), whereas β3-adrenergic agonists elicit potent effects on energy homeostasis by suppressing food intake, and promoting body fat loss, lipid oxidation, oxygen consumption and mitochondrial biogenesis, as well as improving insulin sensitivity and glucose tolerance (Arch and Wilson, 1996; Granneman et al., 2005; Lowell and Spiegelman, 2000; White et al., 2004). Specifically, β3-adrenergic activation stimulates the expression of uncoupling protein-1 (UCP1), a mitochondrial molecule involved in cold-induced nonshivering thermogenesis as well as diet-induced thermogenesis, by uncoupling the respiratory chain in specific adipocytes in both brown (BAT) and white (WAT) adipose tissues (Dallner et al., 2006; Himms-Hagen et al., 2000; Klein et al., 2000; Lowell and Spiegelman, 2000). Thus, chronic β3-adrenergic activation in WAT induces catecholamine-mediated lipolysis, reduces leptin expression in WAT and leptin levels in plasma (Kumar et al., 1999), and elevates adiponectin expression in WAT and adiponectin levels in plasma (Zhang et al., 2002).

On the basis of this pharmacological profile, several clinical trials tried to develop anti-obesity strategies based on β3-adrenergic agonists. The lack of effectiveness in human obesity or the induction of cardiac effects due to the lack of selectivity towards β3-adrenergic receptors led to the withdrawal of these therapies. However, a potentiation of the β3-adrenergic-agonist-induced anti-obesity actions by combinational therapy has now appeared as a new strategy to rescue this type of therapy. As an example, the inhibition of mitogen-activated protein kinase (MAPK) potentiates the effect of β3-adrenergic activation on UCP1-mediated energy dissipation in BAT of fatty acids released from WAT (Inokuma et al., 2006; Klein et al., 2000). In this regard, the stimulation of the transcription factor peroxisome proliferator-activated receptor (PPAR)α, a modulator of appetite and lipid metabolism (Rodríguez de Fonseca et al., 2001), by the natural ligand oleoylethanolamide inhibits insulin-receptor-dependent stimulation of MAPK pathways, providing a frame for a combinational therapy with β3-adrenergic receptor agonists (Martínez de Ubago et al., 2009).

**TRANSLATIONAL IMPACT**

**Clinical issue**

Because of the role of the β3-adrenergic pathway in energy homeostasis, the β3-adrenergic receptor, which is highly expressed in adipose tissue, is a strong candidate for anti-obesity and anti-diabetic therapy. In mice, inhibition of the β3-adrenergic receptor causes obesity, whereas agonists of the receptor elicit anti-obesity effects, such as suppression of food intake and UCP1-mediated thermogenesis. On the basis of these findings, several clinical trials were previously launched to treat obesity using β3-adrenergic agonists. However, like other pharmacological monotherapies devised for the treatment of obesity, these strategies showed disappointingly low levels of clinical efficacy. To circumvent this, research efforts are now focused on the development of polytherapies that exert their effects in different biological processes such as food intake and energy expenditure. An emerging strategy is potentiation of the β3-adrenergic agonist effects by combinatorial therapy.

**Results**

The aim of this study was to determine whether activation of PPARα receptors by oleoylethanolamide (OEA) potentiates the effect of β3-adrenergic stimulation by an agonist, CL316243, in rats. Compared with treatment using CL316243 alone, co-treatment with CL316243 and OEA (CL+OEA) led to a greater reduction in food intake and body weight gain, and a larger increase in energy expenditure. These effects were associated with decreased fat mass and lowered levels of triglycerides in plasma, and with overexpression of thermogenic factors in white adipose tissue. Furthermore, these improved metabolic phenotypes were linked to mitochondrial biogenesis and the appearance of brown fat-like phenotypes in the white adipocytes of CL+OEA-treated rats. Finally, the enhancement of mitochondrial β-oxidation of fatty acids was accompanied by upregulation of *de novo* lipogenesis and a reduction in levels of the unsaturated-fatty-acid-synthesis enzyme *Scd1* in white adipose tissue.

**Implications and future directions**

This study provides evidence that combining β3-adrenergic stimulation with PPARα activation can promote metabolic effects that stimulate energy expenditure, including UCP1-mediated thermogenesis, providing a potential therapeutic approach for the treatment of obesity. The potentiation of β3-adrenergic stimulatory effects by the natural cardioprotective PPARα agonist OEA might also help to lower the effective dose of the β3-adrenergic agonist, reducing the unwanted cardiovascular effects of this class of anti-obesity agent. This study also demonstrates that OEA and CL316243 co-treatment induces white-to-brown adipocyte remodelling. The effects of this remodelling need to be evaluated in humans to allow a definitive prediction of the translational potential of this combinatorial therapy.

In the absence of β3-adrenergic stimulation, differentiation of adipocyte progenitors into white adipocytes in the WAT was dramatically promoted by high-fat feeding (Lee et al., 2012). In contrast, cold exposure or pharmacological activation of β3-adrenergic receptors induces the appearance of brown fat-like (‘brite’) adipocytes in WAT (Cousin et al., 1992; Ishibashi and Seale, 2010; Petrovic et al., 2010), suggesting a mechanism for adipocyte progenitors to promote WAT remodelling. Several data indicate that this process occurs as the result of *de novo* differentiation of stem cells or committed brown preadipocytes (Macotela et al., 2012), or through direct transformation of adult cells via physiological reversible transdifferentiation, which involves genetic reprogramming and tissue reorganization with changes in the density of capillaries and parenchymal nerve fibers (Granneman et al., 2005; Himms-Hagen et al., 2000; Murano et al., 2009). Several transcription factors and coregulators, including PRD1-BF1-RIZ1 homologous domain containing 16 (PRDM16), fibroblast growth factor (FGF)-21, PPARγ coactivator 1α (PGC-1α) and prostaglandins, have been proposed to induce a BAT-specific gene expression profile in response to adrenergic stimulation (Seale et al., 2007; Seale et al., 2008; Seale et al., 2011; Vegiopoulos et al., 2010). Thus, PRDM16 has been identified as a transcriptional coactivator responsible for determining the BAT lineage and has been reported to promote the induction of the thermogenic programme in subcutaneous WAT (Seale et al., 2011). The expression of fibroblast growth factor 21 (FGF21), promoted by β3-adrenergic activation via a p38 MAPK mechanism in BAT, induces higher energy expenditure and body temperature, and triggers a lowering of glucose and triglyceride levels, improved insulin sensitization and enrichment of brown adipocytes (Coskun et al., 2008; Hondares et al., 2011a; Kharitonenkov et al., 2005). Moreover, PPARα is upregulated by the β3-adrenergic receptor agonist CL316243 (Li et al., 2005) and is a critical regulator of thermogenic key components, including PRDM16, FGF21, PGC-1α and UCP1, in BAT (Badman et al., 2007; Chartoumpekis et al., 2011; Hondares et al., 2011a; Hondares et al., 2011b; Villarroya et al., 2007). Thus, PPARα and/or PGC-1α target genes are involved in fatty-acid catabolism, including cellular uptake and mitochondrial and peroxisomal β-oxidation (Mandard et al., 2004), as well as suppression of proinflammatory signalling in adipose tissue (Li et al., 2005).

In the present study, we analyze the potentiation of feeding inhibition, weight loss, thermogenesis and lipid oxidation observed when β3-adrenergic and PPARα receptors are coactivated by the selective adrenergic agonist CL316243 and the natural PPARα receptor ligand OEA, respectively. Because the appearance of ‘brite’ adipocytes within WAT depots is associated with improved metabolic phenotypes, we also explored the expression of mitochondrial (*Cox4i1, Cox4i2*) and BAT (*Prdm16, Fgf21*) factors in epididymal WAT (eWAT) as markers of white-to-brown metaplasia.

## RESULTS

### CL316243 and OEA decreased both feeding and body weight gain

The acute administration of CL316243, BRL37344 and ICI215,001 at 0.1, 0.5 and 1 mg/kg body weight induced significant dose-response reductions of cumulative food intake over 24 hours in rats that had been previously food-deprived for 24 hours (supplementary material Fig. S1A–C). The acute administration of ZD7114, ZD2079 and CGP12177 did not produce any effect or induce an increase of food intake at discrete doses and times over 24 hours (supplementary material Fig. S1D–F). CL316243 at 1 mg/kg was the most effective β3-adrenoceptor agonist and had the most potent dose-response effect on reducing cumulative food intake over time (*P*<0.001 after 24 hours), and this drug and dose were selected for the repeated-treatment experiment.

**Fig. 1:**
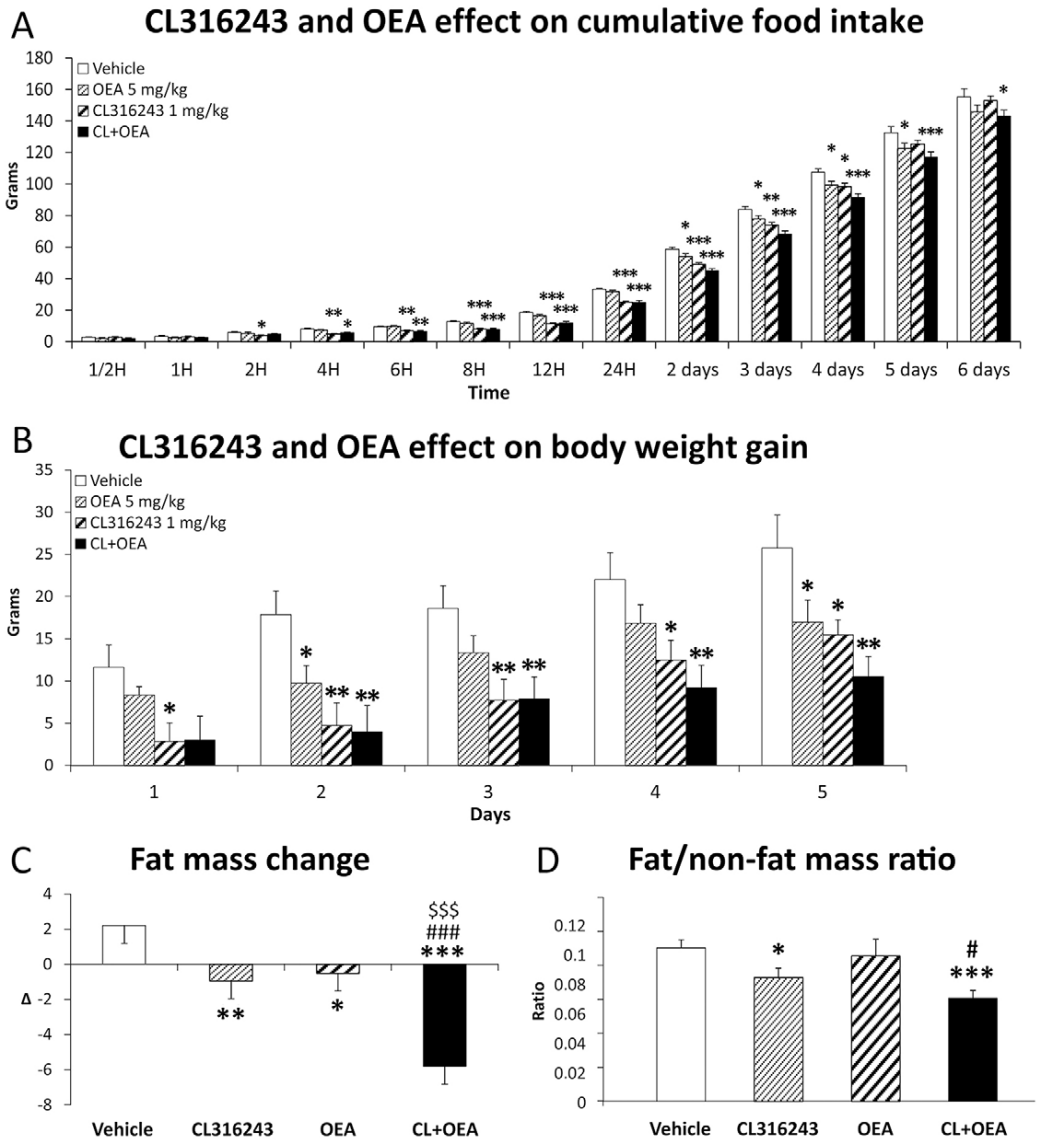
**Effects of repeated administration of CL316243 (1 mg/kg) and/or OEA (5 mg/kg) on food intake, body weight and fat mass over 6 days of treatment.** (A) Cumulative food-intake change over time. (B) Body weight gain change over time. (C,D) Fat mass change and final fat:non-fat mass ratio after 6 days of treatment. Histograms represent the mean±s.e.m. (*n*=8). *P<0.05, **P<0.01, ***P<0.001 versus vehicle-treated rats; ^#^*P*<0.05, ^###^*P*<0.001 versus CL316243-treated rats; ^$$$^*P*<0.001 versus OEA-treated rats.

Over the 6 days of repeated treatment with vehicle, CL316243 (1 mg/kg), OEA (5 mg/kg) and CL316243+OEA (CL+OEA), we monitored cumulative food intake (Fig. 1A; supplementary material Fig. S2). ANOVA analysis showed treatment effects of CL316243, OEA and CL+OEA on cumulative food intake [CL: *F*(1,182)=48.58, *P*<0.0001; OEA: *F*(1,182)=18.13, *P*<0.0001; CL+OEA: *F*(1,182)=96.69, *P*<0.0001]. In all cases, we detected a very significant time effect on cumulative food intake [*F*(12,182)>1313, *P*<0.0001]. OEA did not produce any reduction in cumulative food intake until 2 days of treatment (*P*<0.05), and this reduction disappeared in the 6th day of treatment. Regarding the effect of CL316243, we observed a decrease in cumulative food intake from 2 hours to 4 days of treatment, being very prominent within the first 48 hours (*P*<0.001). *Post hoc* test indicated that the cumulative food intake reduction was more significant and lasting when CL316243 and OEA were co-administered (*P*<0.001 from 6 hours to 5 days; *P*<0.05 after 6 days) than when each was independently administered.

We also monitored the body weight gain over the 6 days of treatment (Fig. 1B). ANOVA indicated that there were treatment effects of CL316243, OEA and CL+OEA on body weight gain [CL: *F*(1,70)=37.11, *P*<0.0001; OEA: *F*(1,70)=13.64, *P*<0.0001; CL+OEA: *F*(1,70)=44.08, *P*<0.0001]. In all cases, we detected a very significant time effect on body weight gain [*F*(4,70) >4.12, *P*<0.005]. OEA induced a slight reduction of body weight gain, being only significant in days 2 and 5 (*P*<0.05). CL316243 showed a decrease in body weight gain from day 2 to day 5, being more prominent within the first 72 hours of treatment (*P*<0.01). Consistent with food intake, the reduction of body weight gain was more significant and lasting when CL316243 and OEA were co-administered (*P*<0.01 from 2 to 5 days of treatment) than when CL316243 and OEA were independently administered.

### CL316243 and OEA synergistically reduced fat mass

We analyzed the change of fat mass and non-fat mass (lean mass) after the repeated treatment of CL316243 and OEA for 6 days (Fig. 1C,E). We detected significant reductions of fat mass after CL316243 (*P*<0.01) or OEA (*P*<0.05) administrations compared with vehicle-treated rats. The fat mass reduction was enhanced after the combined treatment of CL+OEA (*P*<0.001) (Fig. 1C). Interestingly, the ratio of the final fat:non-fat mass confirmed the effects of CL+OEA treatment on fat mass reduction (*P*<0.001) (Fig. 1D).

### CL316243 and OEA enhanced energy expenditure and reduced respiratory VCO_2_/VO_2_ quotient

**Fig. 2:**
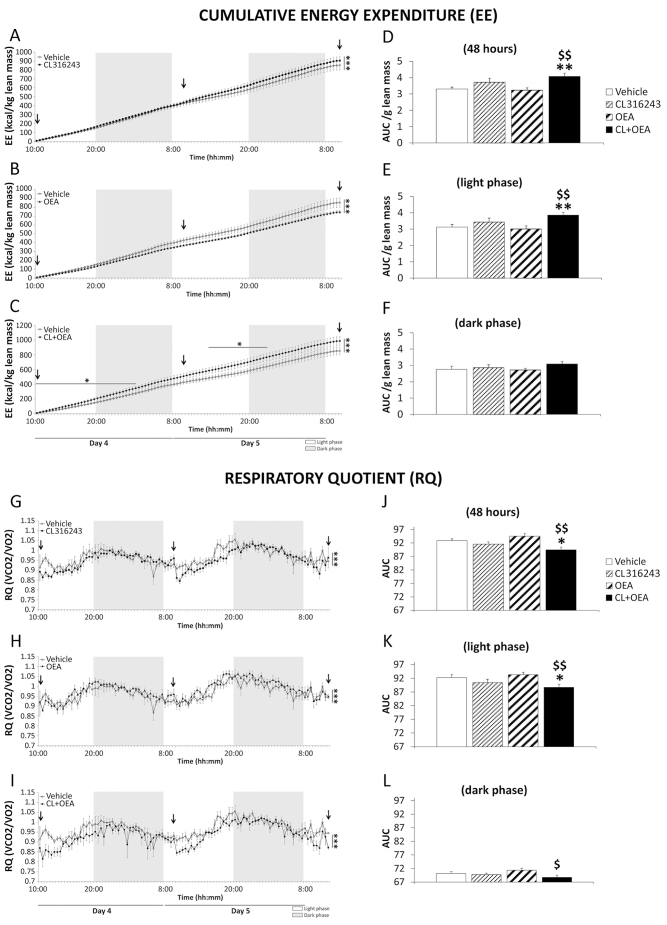
**Effects of repeated administration of CL316243 (1 mg/kg) and/or OEA (5 mg/kg) on energy expenditure and respiratory quotient over 6 days of treatment.** (A–C) Cumulative energy expenditure (EE) for 48 hours (days 4 and 5 of treatment). Arrows indicate the points of administration along time. (D–F) Area under the curve (AUC) of EE for 48 hours and during light and dark phase. (G–I) Respiratory quotient (RQ) for 48 hours (days 4 and 5 of treatment). (J–L) AUC of RQ for 48 hours and during light and dark phase. Histograms and curve points represent the mean±s.e.m. (*n*=8). *P<0.05, **P<0.01, ***P<0.001 versus vehicle-treated rats; ^$^*P*<0.05, ^$$^*P*<0.01 versus OEA-treated rats.

We next evaluated whether CL316243, OEA and CL+OEA treatments induced changes in the other side of the energy-balance equation. CL316243-, OEA- and CL+OEA-treated rats showed significant increases in cumulative energy expenditure (EE) (*P*<0.001) (Fig. 2A–F) and significant decreases in the respiratory quotient (RQ) (*P*<0.001) (Fig. 2G–L). When the area under the curve (AUC) was analyzed, we detected that only the combination of CL+OEA treatment induced significant EE increase (*P*<0.01) (Fig. 2D–F) and RQ decrease (*P*<0.05) (Fig. 2J–L) compared with vehicle-treated rats. Both effects of CL+OEA on EE and RQ were mainly produced during the light phase. Cumulative locomotor activity (LA) was not modified after CL+OEA treatment (supplementary material Fig. S3). When the AUC was analyzed, a significant LA increase was detected after OEA treatment (*P*<0.05) that was blocked by CL316243 (*P*<0.05) during the dark phase.

### CL316243 and OEA increased BAT temperature, which was accompanied with higher BAT weight

**Fig. 3:**
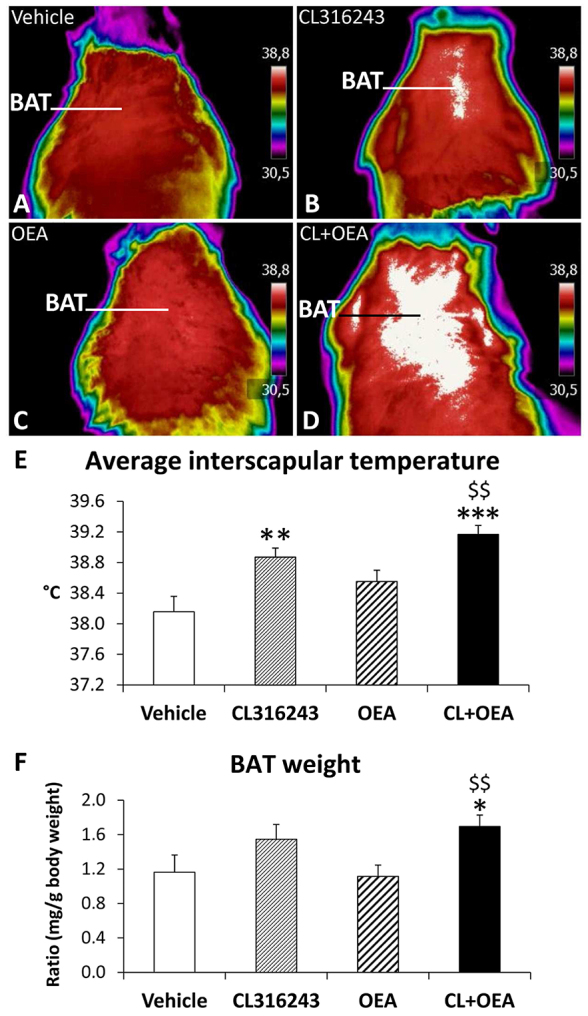
**Effects of repeated administration of CL316243 and/or OEA on BAT temperature and weight after 6 days of treatment.** (A–D) Representative infrared thermal images with quantification of interscapular temperature. Histograms represent the mean±s.e.m. (*n*=8). (E) Average interscapular temperature. (F) BAT weight. *P<0.05, **P<0.01, ***P<0.001 versus vehicle-treated rats; ^$$^*P*<0.01 versus OEA-treated rats.

Whereas OEA did not affect the temperature surrounding interscapular BAT, CL316243 induced an increased temperature (Fig. 3A–E). However, co-administration of CL+OEA showed a more potent increase in the temperature surrounding interscapular BAT compared with that of vehicle-treated rats (*P*<0.01 and *P*<0.001, respectively) (Fig. 3A–E). Consistently, only the increase of BAT temperature after the co-administration of CL+OEA was accompanied with an increase in the amount of BAT weight (*P*<0.05) (Fig. 3F).

### CL316243 and OEA increased the expression of PPAR and UCP1 in eWAT and BAT, and reduced p38 MAPK phosphorylation in eWAT

To obtain information about the regulators mediating CL+OEA-induced negative energy balance and BAT temperature, first we examined the morphology of the white and brown adipocytes, and then we analyzed the expression of thermogenic markers such as PPARα and UCP1 in eWAT and BAT. Haematoxylin and eosin staining showed clear differences in the morphology of the white and brown adipocytes in a treatment-dependent manner (supplementary material Fig. S4A–B′). In the white adipocytes, CL316243 and CL+OEA treatments produced a dramatic reduction in the size of the monolocular lipid droplet and, as a consequence, an increase of the cytoplasm appearance (supplementary material Fig. S4A–D). In the brown adipocytes, CL316243 induced a higher fragmentation of the multilocular lipid droplets, whereas OEA and CL+OEA treatments produced almost total removal of lipid droplets (supplementary material Fig. S4A′–D′). The dramatic change after CL+OEA treatment made the morphology and limits of the brown adipocytes unrecognizable (supplementary material Fig. S4D,D′).

In keeping with these effects, CL316243 and CL+OEA treatments for 6 days increased the expression of PPARα and UCP1 immunofluorescence in eWAT (*P*<0.001 and *P*<0.05, respectively) (supplementary material Fig. S4E,J). In eWAT and BAT, we observed an increase of PPARα immunofluorescence after CL316243 and CL+OEA treatments (*P*<0.05 and *P*<0.01, respectively) (supplementary material Fig. S4E′), but only after CL+OEA coadministration did we detect an increase of UCP1 immunofluorescence in BAT (*P*<0.05) (supplementary material Fig. S4J′).

**Fig. 4:**
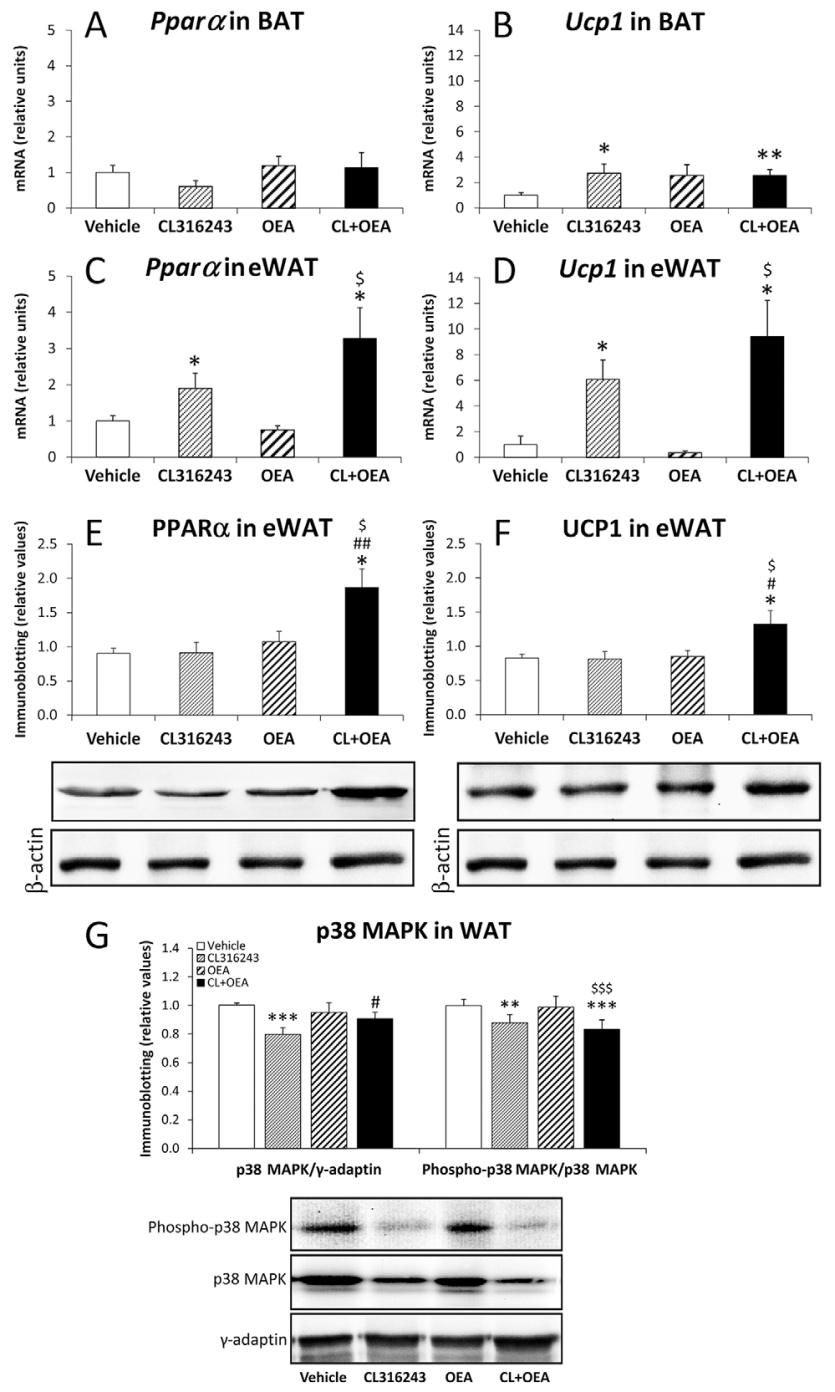
**Effects of repeated administration of CL316243 and/or OEA on expression of molecules implicated in thermogenesis (*Pparα* and *Ucp1*) in eWAT and BAT, and on protein expression of p38 MAPK and p38 MAPK phosphorylation in eWAT after 6 days of treatment.** (A,B) *Pparα* and *Ucp1* mRNA levels in BAT. (C,D) *Pparα* and *Ucp1* mRNA levels in eWAT. (E-G) Protein levels of PPARα, UCP1, p38 MAPK and phosphorylated p38 MAPK in eWAT. Histograms represent the mean±s.e.m. (*n*=8). *P<0.05, **P<0.01, ***P<0.001 versus vehicle-treated rats; ^#^*P*<0.05, ^##^*P*<0.01 versus CL316243-treated rats; ^$^*P*<0.05, ^$$$^*P*<0.001 versus OEA-treated rats.

The effect of CL+OEA treatment on the increase of PPARα and UCP1 immunofluorescences in eWAT was confirmed after the analysis of the gene and protein expressions by quantitative realtime reverse transcription polymerase chain reaction (RT-qPCR) and western blot techniques. Thus, increased levels of *UCP1* mRNA, but not *PPARα,* were detected in the BAT only after CL316243 and CL+OEA treatments (*P*<0.01 and *P*<0.05, respectively) (Fig. 4A,B). Increased levels of *PPARα* and *UCP1* mRNA were observed in the eWAT of CL316243-and CL+OEA-treated rats (*P*<0.05) (Fig. 4C,D). Moreover, CL+OEA co-administration also produced increased protein levels of PPARα and UCP1 in WAT (*P*<0.05) compared with vehicle-treated rats (Fig. 4E,F).

We evaluated whether CL316243, OEA and CL+OEA effects on PPARα and UCP1 were conducted by a p38 MAPK-dependent activation. Decreased protein levels of p38 MAPK was observed in the WAT of CL316243-treated rats (*P*<0.001), but this effect was blocked when CL+OEA was co-administered (Fig. 4G). Interestingly, p38 MAPK phosphorylation was also reduced in the WAT of CL316243-treated rats (*P*<0.01), but this effect was enhanced when CL+OEA was co-administered (*P*<0.001) (Fig. 4G).

### CL316243 and OEA potentiated the expression of both mitochondrial and BAT markers in eWAT

**Fig. 5:**
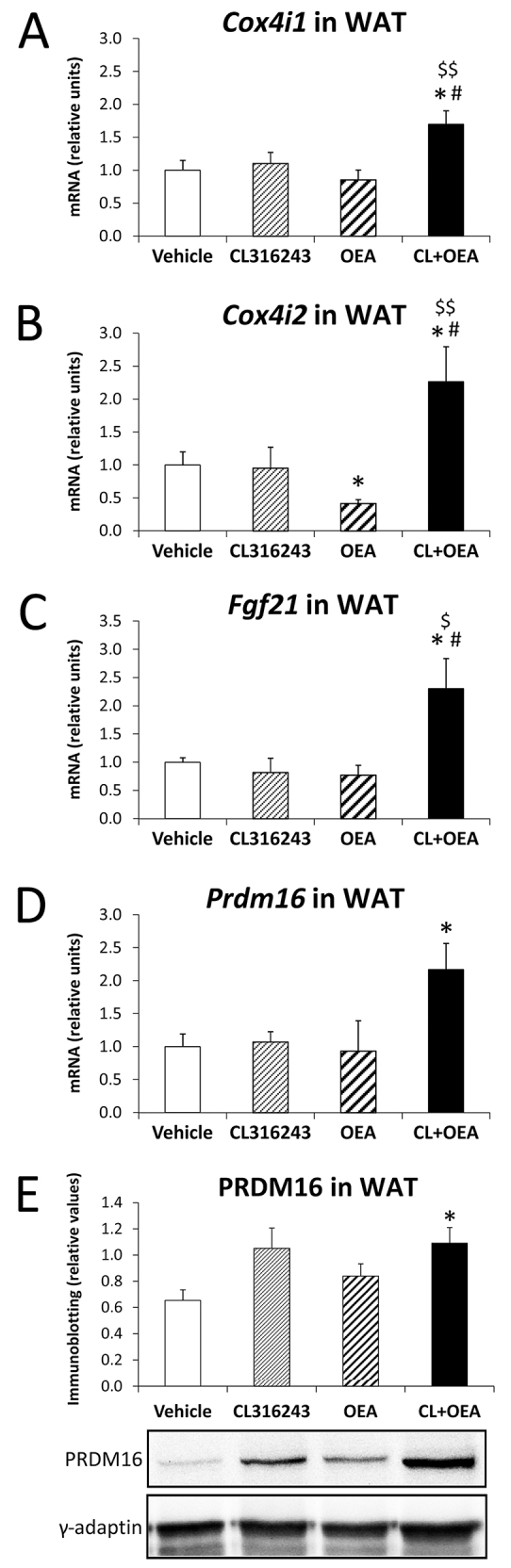
**Effects of repeated administration of CL316243 and/or OEA on gene expression of molecules implicated in mitochondrial programmes (*Cox4i1* and *Cox4i2*) and the BAT-like phenotype (*Fgf21* and *Prdm16*) in eWAT after 6 days of treatment.** (A–D) *Cox4i1*, *Cox4i2*, *Fgf21* and *Prdm16* mRNA levels in eWAT. (E) PRDM16 protein levels in eWAT. Histograms represent the mean±s.e.m. (*n*=8). *P<0.05 versus vehicle-treated rats; ^#^*P*<0.05 versus CL316243-treated rats; ^$^*P*<0.05, ^$$^*P*<0.01 versus OEA-treated rats.

**Fig. 6:**
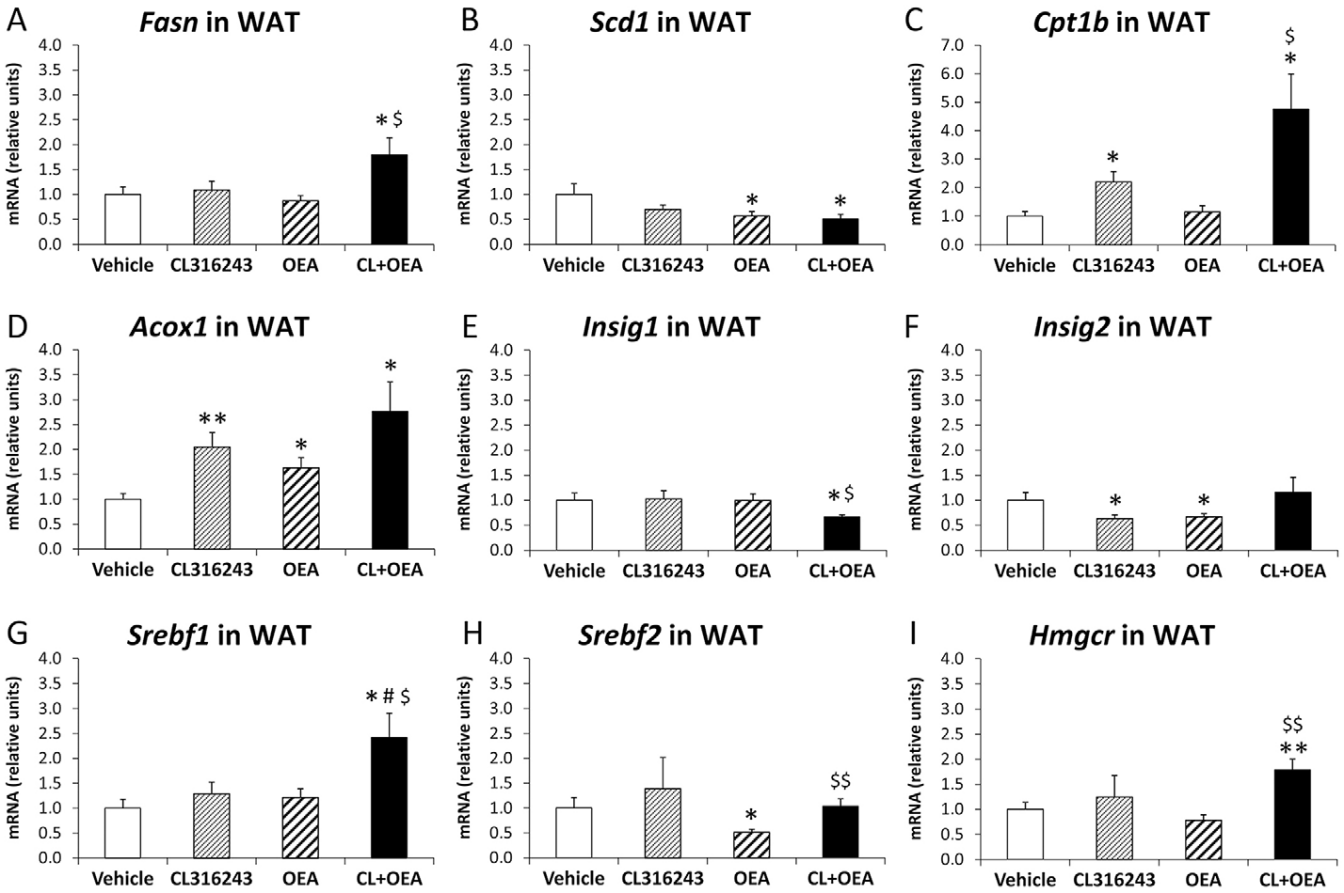
**Effects of repeated administration of CL316243 and/or OEA on gene expression of molecules implicated in lipid and cholesterol metabolism and fatty acid β-oxidation in WAT after 6 days of treatment.** (A–I) Quantification of mRNA levels of *Fasn*, *Scd1*, *Cpt1b*, *Acox1*, *Insig1*, *Insig2*, *Srebf1*, *Srebf2* and *Hmgcr* in WAT. Histograms represent the mean±s.e.m. (*n*=8). *P<0.05, **P<0.01 versus vehicle-treated rats; ^#^*P*<0.05 versus CL316243-treated rats; ^$^*P*<0.05, ^$$^*P*<0.01 versus OEA-treated rats.

To obtain information about the regulators promoting CL+OEA-induced BAT-like features in eWAT, we analyzed the expression of mitochondrial markers such as the cytochrome *c* oxidase subunit 4 isoform 1 and 2 (Cox4i1 and Cox4i2), and BAT markers such as FGF21 and PRDM16. CL+OEA co-administration induced increased expression of *Cox4i1*, *Cox4i2*, *Fgf21* and *Prdm16* mRNA in eWAT (all at *P*<0.05) (Fig. 6A–D). The increase of *Prdm16* gene expression in eWAT after CL+OEA treatment was confirmed when protein levels of PRDM16 were analyzed (*P*<0.05) (Fig. 5E,F).

### CL316243 and OEA modulated lipid- and cholesterol-metabolism-related factors and increased D -oxidation-related factors in eWAT

To investigate whether the reduction of fat mass and droplets in WAT involves an alteration in lipid and cholesterol metabolism, we analyzed the gene expression of factors related to fatty acid synthesis such as *Fasn* and *Scd1*, β-oxidation such as *Cpt1b* and *Acox1*, and cholesterol synthesis such as *Insig1*, *Insig2*, *Srebf1*, *Srebf2* and *Hmgcr*. The WAT of CL316243-treated rats showed increased levels of *Cpt1b* and *Acox1* mRNA (*P*<0.05 and *P*<0.01, respectively) (Fig. 6C,D) and a decreased level of *Insig2* mRNA (*P*<0.05) (Fig. 6F) compared with vehicle-treated rats. OEA-treated rats showed an increased level of *Acox1* mRNA (*P*<0.05) (Fig. 6D) but decreased levels of *Scd1*, *Insig2* and *Srebf2* (*P*<0.05) (Fig. 6B,F,H). The co-administration of CL+OEA produced increased expressions of *Fasn*, *Cpt1b*, *Acox1*, *Srebf1* and *Hmgcr* mRNA (all at *P*<0.05, except *Hmgcr* at *P*<0.01) (Fig. 6A,C,D,G,I) but decreased expressions of *Scd1* and *Insig1* mRNA (*P*<0.05) (Fig. 6B,E) compared with vehicle-treated rats.

### CL316243 and OEA decreased plasma triglycerides, total cholesterol, NEFAs, leptin and TNF-, but increased plasma glucose and the fT_3_:fT_4_ ratio

To characterize the metabolic state in the plasma of CL316243-, OEA- and CL+OEA-treated rats, we evaluated the plasma levels of relevant metabolites such as glucose, triglycerides, total cholesterol, high-density lipoprotein (HDL)-cholesterol and non-essential fatty acids (NEFAs), several metabolic hormones such as insulin and the free (unbound) and active thyroid hormones free triiodothyronine (fT3) and free thyroxine (fT_4_), and several adipokines such as leptin, adiponectin, interleukin-6 (IL-6), interleukin-1β (IL-1β) and tumour necrosis factor-α (TNF-α). CL316243-treated rats showed reduced plasma levels of NEFAs (*P*<0.05) and a lowered insulin resistance (*P*<0.05) after using the homeostatic model assessment (HOMA-IR) (Table 1). In contrast, CL316243 induced a significantly higher fT_3_:fT_4_ ratio (*P*<0.001) (Table 1). The plasma of OEA-treated rats showed reduced levels of total cholesterol, but higher levels of insulin and fT_3_:fT_4_ ratio compared with vehicle-treated rats (all at *P*<0.05) (Table 1). The co-administration of CL+OEA induced significant increases of glucose (*P*<0.01), HDL-cholesterol and fT3:fT4 ratio (*P*<0.05), but significant decreases of triglycerides (*P*<0.01), total cholesterol (*P*<0.01), NEFAs (*P*<0.05) and HOMA-IR (*P*<0.05) compared with the vehicle group (Table 1).

**Table 1:**
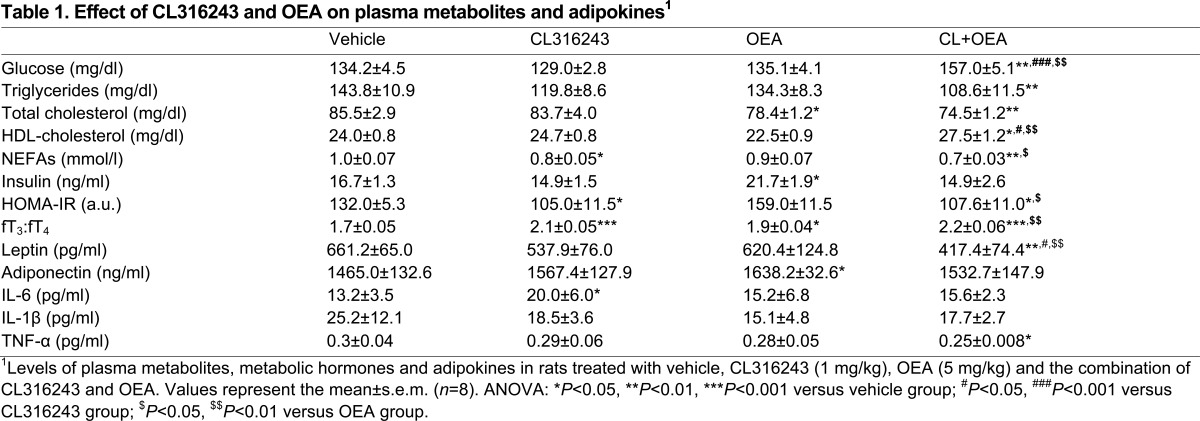
Effect of CL316243 and OEA on plasma metabolites and adipokines^1^

Regarding adipokine levels, CL316243 increased the plasma levels of IL-6 (*P*<0.05), and OEA increased the plasma levels of adiponectin (*P*<0.05), but CL+OEA reduced the plasma levels of leptin (*P*<0.01) and TNF-α (*P*<0.05) (Table 1).

## DISCUSSION

**Fig. 7:**
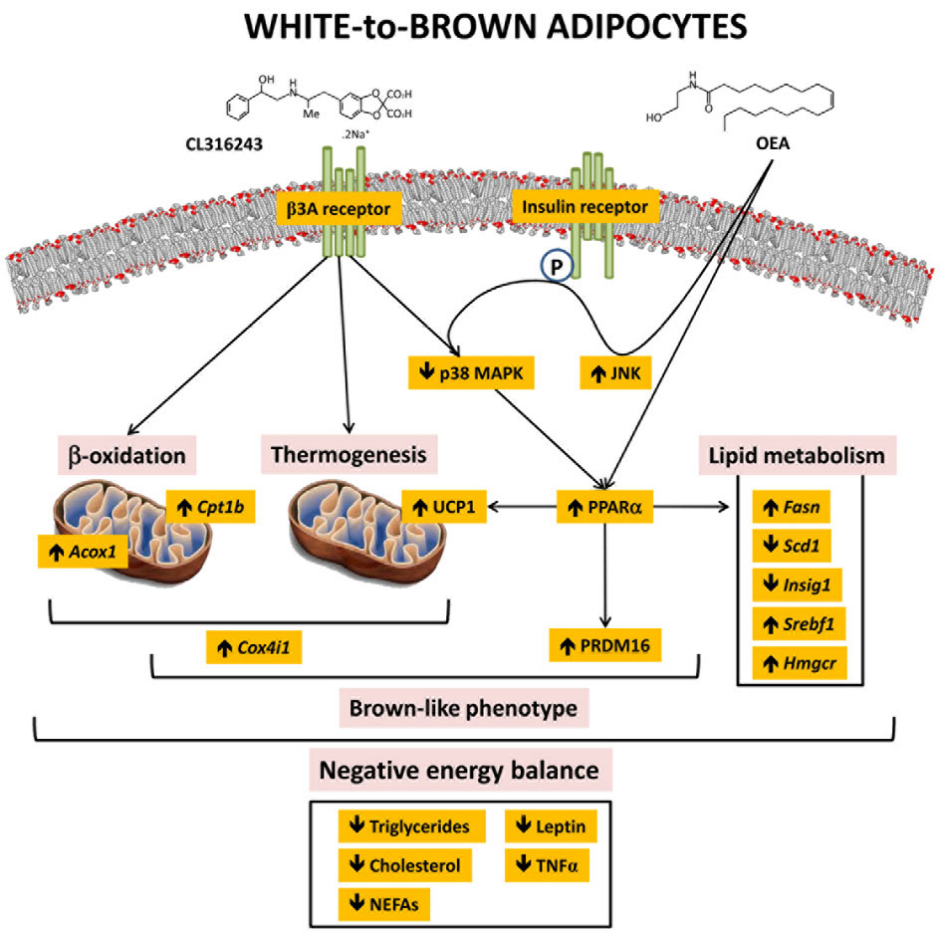
**Model of the regulation of thermogenesis, β-oxidation and lipid metabolism by the co-adjuvant activation of β3-adrenergic and PPARα receptors.**

Considering the energy-dissipating ability of UCP1 by β3-adrenergic stimulation, the enhancement of its expression and activation by transcription factors and coactivators can be a promising strategy for anti-obesity and anti-diabetic drug therapy. A combinatorial therapy based on the association of β3-adrenergic receptor stimulation with the natural PPARα receptor ligand OEA was developed on the basis of potential convergent effects of OEA with the adrenergic mechanisms in the adipose tissue. Thus, the aim of the present study was to determine whether PPARα activation potentiates the effect of β3-adrenergic stimulation on energy balance in association with UCP1-mediated thermogenesis, white-to-brown adipocyte metaplasia and reduced fat content. We examined several factors that are crucial for the regulation of thermogenesis, fatty acid β-oxidation and lipid metabolism, as well as the promotion of mitochondrial and BAT-like features in eWAT. The principal findings in the present study were as follows (Fig. 7): (1) CL316243 and OEA potentiated the reduction of food intake and body weight gain and increased energy expenditure. (2) The reduced body weight was accompanied with decreased fat mass and lowered levels of triglycerides, cholesterol, NEFAs, leptin and TNF-α in plasma. (3) All these effects can be linked with the overexpression of the thermogenic factors UCP1 and PPARα in eWAT and BAT, which, in turn, decreases p38 MAPK phosphorylation in eWAT. (4) These improved metabolic phenotypes concurred with mitochondrial biogenesis and the appearance of brown fat-like phenotypes in the white adipocytes: the mitochondrial (*Cox4i1, Cox4i2*) and BAT (*Fgf21, Prdm16*) genes were overexpressed in eWAT of CL+OEA-treated rats. (5) Finally, regarding the mitochondrial β-oxidation of fatty acids and lipid metabolism, the enhancement of *Cpt1b* and *Acox1* was accompanied with an upregulation of *de novo* lipogenesis and a reduction of the unsaturated-fatty-acid-synthesis enzyme *Scd1* in eWAT (Fig. 7).

Our results indicated that there were gene and protein expression changes in eWAT based on three levels of activation: (1) specific changes in *PPARα, Ucp1* and *Cpt1b* mRNA levels and phospho-p38 MAPK protein levels in eWAT based on β-adrenergic activation. Regarding this issue, three facts should be noted: (a) the increased gene expressions of *PPARα, Ucp1* and *Cpt1b* were enhanced with the co-administration of the PPARα agonist OEA, and (b) the increased protein levels of PPARα and UCP1 were only detected after PPARα and β-adrenergic coactivation. (c) The increased level of p38 MAPK phosphorylation in eWAT by β-adrenergic stimulation was also enhanced by PPARα coactivation. These results suggest that, in some way, PPARα activation produced a synergic effect downstream on the β-adrenergic pathway in the adipose tissue. (2) Specific changes in *Scd1* mRNA levels based on PPARα activation. (3) Synergic changes based on the coactivation of β-adrenergic and PPARα receptors that mainly produced an overexpression of the mitochondrial (*Cox4i1*, *Cox4i2*) and BAT (*Fgf21*, *Prdm16*) genes, and the β-oxidation (*Acox*) and the lipid metabolism (*Fasn*, *Srebf1* and *Hmgcr*) genes in eWAT. Altogether, the three levels of activation produced convergent effects of OEA with the adrenergic mechanisms in the adipose tissue.

The involvement of UCP1 in adrenergically induced thermogenesis demonstrates that UCP1 plays a significant role in the control of energy expenditure; its dysfunction contributes to the development and maintenance of obesity (Feldmann et al., 2009; Inokuma et al., 2006; Ricquier and Bouillaud, 2000). Our data indicated that PPARα and β3-adrenergic coactivation potentiated the decrease of body weight through a decrease in food intake and increased energy expenditure. According to the increased energy expenditure and decreased fat mass, we found elevated interscapular temperature, which was explained by stimulated UCP1 expression in eWAT and BAT. Furthermore, we also detected a reduced insulin resistance and lowered levels of triglycerides, cholesterol and NEFAs in CL+OEA-treated rats. Body composition and histological data indicated that the decrease in fat mass, the reduction of fat depots and/or the change from monolocular to multilocular lipid droplets in the white adipocytes were correlated with decreased levels of leptin and TNF-α in plasma of CL+OEA-treated rats. Moreover, CL+OEA treatment seemed to reduce dramatically the levels of multilocular lipid droplets that characterize brown adipocytes in BAT. Previous studies reported that leptin secretion is positively correlated with adiposity (Skurk et al., 2007), and that TNF-α action is implicated in inflammatory response and insulin resistance, at least in obesity (Zhang et al., 2002). Regarding eWAT, these results agree with the fact that CL316243 stimulated PPARα expression and fatty acid β-oxidation (*Cpt1b* and *Acox1*) and reduced the expression of *Scd1*, a critical control point of lipid partitioning, obesity development and diet-induced hepatic insulin resistance (Gutiérrez-Juárez et al., 2006; Jiang et al., 2005). Interestingly, this metabolic response was potentiated by the lipolytic effect of OEA (Guzmán et al., 2004; Rodríguez de Fonseca et al., 2001), whereby this elevated capacity for β-oxidation within WAT contributes to insulin-sensitizing effects (lowering insulin resistance) and reduced proinflammatory signalling (lowering TNF-α level in plasma). Taken together, all these effects clearly contribute to anti-obesity actions.

Improved metabolic rates are associated with the appearance of brown fat-like (‘brite’) phenotypes within eWAT depots. UCP1 expression and uncoupling respiration is highly induced by transcription factors related to brown fat-like multilocular adipocytes such as PRDM16, FGF21 and/or PPARα and PGC-1α, among others (Seale et al., 2007; Seale et al., 2011; Hondares et al., 2011a; Hondares et al., 2011b). Thus, transgenic expression of PRDM16 in white adipocytes stimulates the formation of brown fat-like adipocytes (Seale et al., 2007). Our findings strongly indicate that β3-adrenergic stimulation of UCP1 expression in eWAT and BAT seems not to require the participation of PPARα, but UCP1 expression can be enhanced after the administration of the PPARα ligand OEA. This effect of the β3-adrenergic receptor controlling UCP1 expression can be regulated by p38 MAPK mechanisms (Klein et al., 2000), because phosphorylated levels of p38 MAPK were reduced in eWAT of CL316243-treated rats, with this effect being more prominent after CL+OEA treatment. In contrast, the coactivation of PPARα and β3-adrenergic receptors is most likely required for the expression of PRDM16, FGF21 and the mitochondrial factors *Cox4i1* and *Cox4i2* in eWAT. These features seem to be related to the promotion of transdifferentiation towards brown fat-like adipocytes, including mitochondrial biogenesis, in eWAT (Himms-Hagen et al., 2000; Villarroya et al., 2007). However, chronic and systemic administration of these selective β3-adrenergic and PPARα agonists seems to be insufficient to induce PRDM16 and FGF21 expression in BAT, suggesting that either participation of additional coactivators such as PGC-1α, highly expressed in B AT (Barbera et al., 2001), or the stimulus driven by cold acclimatization, high-fat feeding or higher free-fatty-acid mobilization from triglyceride stores, could be necessary in this process (Chartoumpekis et al., 2011; Seale et al., 2011). The latter hypothesis is in harmony with the fact that there is differential sensitivity in sympathetic stimulation of WAT and BAT. Thus, BAT-specific β3-adrenergic transgenic re-expression into β3-adrenergic knockout mice failed to rescue CL316243-mediated effects on food intake and minimally restored effects on oxygen consumption, indicating that a full stimulation required the presence of β3-adrenergic receptors in white adipocytes (Grujic et al., 1997).

Besides the peripheral β3-adrenergic mechanisms on sympathetic activity, there is a role for β3-adrenergic receptors in the central control of feeding. Intracerebroventricular administration of β3-adrenergic agonists causes a dose-dependent decrease in food intake (Tsujii and Bray, 1992) and affects neurons within specific hypothalamic nuclei such as paraventricular, lateral, ventromedial and dorsal hypothalamic nuclei (Castillo-Meléndez et al., 2000). Interestingly, OEA, by its ability to engage PPARα (Fu et al., 2003), is involved in the peripheral regulation of feeding. OEA can activate sensory vagus fibers, which in turn inhibit feeding by the discrete activation of the paraventricular hypothalamic nucleus and the nucleus of the solitary tract. However, OEA does not affect food intake when injected into the brain ventricles (Rodríguez de Fonseca et al., 2001; Schwartz et al., 2008). Further analysis is needed to find direct evidence of β3-adrenergic activation in the CNS. However, we observed some CL316243-mediated effects that can be indirectly related with a possible central action. Thus, the dose- and time-related decrease in food intake after intraperitoneal (i.p.) CL316243 administration was similar to those described in previous studies after ventricular infusions of β3-adrenergic agonists (Tsujii and Bray, 1992), suggesting similar sympathetic and/or central β3-adrenergic activation. The changes in food intake are closely related by the central control of appetite and feeding behaviour processes (affective and mnemonic aspect of eating) in the hypothalamus and hippocampus. Additionally, the alterations of leptin and TNFα levels in plasma can affect the central regulation of energy balance.

The observation that UCP1-mediated thermogenesis through β3-adrenergic activation is required for maximal stimulation of energy expenditure have important implications for the treatment of human obesity. Given that BAT has traditionally been considered to show discrete physiological relevance in humans (Cannon and Nedergaard, 2004; Virtanen et al., 2009), it is reasonable to anticipate that the effect of β3-adrenergic agonists on energy expenditure in humans will be less efficient than might be expected. However, several data point to a higher physiological relevance of β3-adrenergic stimulation when human white adipocytes acquire functional features (UCP1) of brown fat-like phenotypes (Cinti, 2009; Oberkofler et al., 1997; Tiraby et al., 2003). Whether the proposed combinational therapy tested in the present study works in human adipose tissue remains to be elucidated. It might be important to know whether human adipose tissue will be sensitized to the anti-obesity actions of β3-adrenergic agonists by the actions of OEA and PPARα receptor agonists.

In summary, the present results support the utility of β3-adrenergic receptor agonist-based combinational therapies for future therapeutic strategies to treat human obesity. The present study demonstrates that this combinational therapy promotes adipocyte remodelling in eWAT. This study not only support the roles of co-stimulation of β3-adrenergic and PPARα receptors on the induction of white-to-brown adipocyte phenotypes, but also set the place for the utility of new regulators such as PRDM16 for the development of new therapeutics of complicated obesity. In any event, our data showing that combined administration of OEA and β3-adrenergic agonists show clear-cut effects in body weight, body composition and different metabolic markers open up the avenue for translational studies in their therapeutic use in obesity.

## MATERIALS AND METHODS

### Ethics statement

All experimental procedures with animals were performed in compliance with the European Communities directive of 24 November 1986 (86/609/ECC) and Spanish legislation (BOE 252/34367-91, 2005) regulating animal research. Research procedures included in the present study were approved by the Research and Bioethics Committee of University of Santiago de Compostela and Hospital Carlos Haya. For all the analytic methods, we used eight rats per experimental group.

### Animals and housing

Adult male Sprague-Dawley rats that weighed ~300 g at the beginning of the experiments (Animal House, University of Santiago de Compostela) were used in this study. All animals were experimentally naïve, and they were individually housed in controlled room conditions (temperature: 22±2°C; humidity: 40±5%; 12-hour light-dark cycle, lights on at 8:00 am) with free access to food and tap water. The cumulative food intake by each rat and their body weight gain were monitored throughout the experiments.

### Drugs

To select the most effective β3-adrenoceptor agonist for the chronic study, several drugs were tested in a dose-response study. The potent and highly selective β3 agonists CL316243 disodium salt {5-[(2*R*)-2-[[(2*R*)-2-(3-chlorophenyl)-2-hydroxyethyl]amino]propyl]-1,3-benzodioxole-2,2-dicarboxylic acid, disodium salt} (cat. no. 1499, Tocris Bioscience, Bristol, UK) and BRL37344 sodium salt {(R*,R*)-(±)-4-[2-[(2-(3-chlorophenyl)-2-hydroxyethyl)amino]propyl]phenoxyacetic acid, sodium salt} (cat. no. 0948, Tocris Bioscience), and the less potent β3 agonists ICI215,001 hydrochloride {(*S*)-4-[2-hydroxy-3-phenoxypropylaminoethoxy] phenoxyacetic acid hydrochloride} (cat. no. 0929, Tocris Bioscience), ZD7114 hydrochloride {(*S*)-4-[2-hydroxy-3-phenoxypropylaminoethoxy]-*N*-(2-methoxyethyl) phenoxyacetamide hydrochloride} (cat. no. 0930, Tocris Bioscience) and ZD2079 {4-[2-[[(2R)-2-hydroxy-2-phenylethyl]amino]ethoxy]-benzeneacetic acid hydrochloride} (cat. no. 2154, Tocris Bioscience) were used. We also tested the partial β3 agonist CGP12177 {4-[3-[(1,1-dimethylethyl)amino]2-hydroxypropoxy]-1,3-dihydro-2*H*-benzimidazol-2-one hydrochloride} (cat. no. 1134, Tocris Bioscience). All these drugs were dissolved in sterile saline (0.9% NaCl) and administered i.p. at doses of 0.1, 0.5 and 1 mg/kg body weight. The natural PPARα agonist oleylethanolamide [OEA, (9Z)-N-(2-hydroxyethyl)-9-octadecenamide] (cat. no. 1484, Tocris Bioscience) was dissolved in 5% Tween 20 and sterile saline and administered i.p. at a dose of 5 mg/kg body weight.

### Treatment

For acute dose-response treatment, rats received one i.p. injection of either vehicle (1 ml/kg body weight of 5% Tween 20 in sterile saline) or the β3 agonists CL316243, BRL37344, ICI215,001, ZD7114, ZD2079 and CGP12177 at 0.1, 0.5 and 1 mg/kg body weight. For repeated treatment, rats received a daily i.p. injection either of vehicle (1 ml/kg body weight of 5% Tween 20 in sterile saline), CL316243 at 1 mg/kg body weight and/or OEA at 5 mg/kg body weight over 6 days. Food and water remained unchanged and *ad libitum.* Finally, we generated four experimental groups for acute treatment with either of the above β3 agonists (*n*=8): vehicle, 0.1 mg/kg, 0.5 mg/kg and 1 mg/kg body weight; and four experimental groups for repeated treatment with CL316243 and OEA (*n*=8): vehicle, CL316243 1 mg/kg, OEA 5 mg/kg body weight and the combination of CL316243 and OEA.

### Measurement of food intake and body weight

After one administration of either of the above β3 agonists at 0.1, 0.5 or 1 mg/kg body weight (acute dose-response treatment), the cumulative food intake was measured over a time course of 0.5, 1, 2, 4, 6, 8, 12 and 24 hours in rats previously food-deprived for 24 hours. The optimal β3 agonist and dose at which treatment would be more effective on food intake changes were selected for the repeated treatment experiment. During repeated treatment of CL316243 at 1 mg/kg and OEA at 5 mg/kg body weight (Rodríguez de Fonseca et al., 2001; White et al., 2004), we measured the cumulative food intake and the body weight gain every day during the 6 days of treatment.

### Measurement of energy expenditure, respiratory quotient and locomotor activity

During 48 hours from the fourth day of the repeated treatment experiment, rats were analyzed for energy expenditure (EE, kcal/kg lean mass), respiratory quotient (RQ, VCO_2_/VO_2_), food intake and locomotor activity (LA) using a calorimetric system (LabMaster, TSE System, Bad Homburg, Germany) (Imbernon et al., 2013). This system is an open-circuit instrument that determines: (1) the energy consumed by the amount of caloric intake (kilocalories) along time (hours) and normalized by the lean mass (kilograms); (2) the ration between the CO_2_ production and O_2_ consumption (VCO_2_/VO_2_); and (3) the total horizontal locomotion. Previously, all rats were acclimated to the experimental room and habituated to the system for 48 hours before starting the measurements.

### Measurement of body composition

We analyzed the variation of the amount of fat mass and non-fat mass (lean mass) before and after the 6-day treatment of CL316243 and OEA using a nuclear magnetic resonance imaging (Whole Body Composition Analyzer, EchoMRI, Houston, TX) (Imbernon et al., 2013).

### Measurement of temperature

Interscapular temperature surrounding BAT was recorded with an infrared camera (Compact-Infrared-Thermal-Imaging Camera E60bx, FLIR, West Malling, Kent, UK) and analyzed with a specific software package (FLIR Tools Software). For each animal/group (*n*=8), three or four pictures were taken and analyzed. The temperature surrounding BAT for one particular animal was calculated as the average temperature in a defined interscapular area (2 cm Ø) recorded by analyzing those pictures.

### Sample collection

Animals from the four repeated treatment experimental groups (*n*=8; vehicle, CL316243, OEA and CL316243+OEA) were killed by decapitation 2 hours after the last dose of treatment in a separate room from the other experimental animals. Blood samples were briefly collected and centrifuged (1000*g* for 10 minutes at 4°C), and all plasma samples were frozen at −80°C for biochemical and hormonal analysis. White (epididymal fat, eWAT) and brown (BAT) adipose tissues and liver were dissected out. Part of each sample was fixated with 4% paraformaldehyde in 0.1 M phosphate buffered saline (PBS) by immersion until immunohistochemical analysis. The remaining of each sample was briefly frozen at −80°C until RT-qPCR and western blot analyses.

### Measurement of metabolites, metabolic hormones and adipokines in plasma

Blood samples from rats treated with vehicle, CL316243, OEA and CL+OEA for 6 days were collected into tubes containing EDTA-2Na (1 mg/ml blood). The samples were immediately centrifuged, and the plasma aliquoted and stored at −80°C until the determination of biochemical parameters. The following metabolites, metabolic hormones and adipokines were measured in plasma: glucose, triglycerides, total cholesterol, high-density lipoprotein (HDL)-cholesterol, NEFAs, insulin, fT_3_, fT_4_, leptin, adiponectin, IL-6, IL-1β and TNF-α. The metabolites were analyzed using commercial kits according to the manufacturer’s instructions, and a Hitachi 737 Automatic Analyzer (Hitachi Ltd, Tokyo, Japan). The insulin levels were measured using a commercial rat insulin ELISA kit (cat. no. 10-1113-01, Mercodia, Sweden). The free (unbound) and active thyroid hormones were measured using commercial Advia Centaur® fT_3_ and fT_4_ Ready Packs assays for direct chemiluminescence in an Advia Centaur® XP immunoassay system (Siemens, Erlangen, Germany). The adipokines were analyzed using a commercial rat adipocyte Millipex™ Map kit (cat. no. RADPCYT-82K, Millipore, Billerica, MA) and a Luminex 100™ IS v.1.7 system (Luminex, Austin, TX).

### Liver fat extraction and content

We performed fat extraction as was described previously (Alonso et al., 2012). Total lipids were extracted from frozen liver samples with chloroform-methanol (2:1, v/v) and butylated hydroxytoluene (0.025%, w/v) according to the Bligh and Dyer method. After two centrifugation steps (2800*g*, 4°C for 10 minutes), the lower phase containing lipids was extracted with a Pasteur pipette. Nitrogen was used to dry each sample, and the liver fat content was expressed as a percentage of the tissue weight (supplementary material Table S1).

### RNA isolation and RT-qPCR analysis

We performed real-time PCR (TaqMan, Applied Biosystem, Carlsbad, CA) as described previously (Crespillo et al., 2011a; Decara et al., 2012) using specific sets of primer probes (supplementary material Tables S2, S3). Briefly, tissue portions of eWAT and BAT (~100 mg) were homogenized on ice and RNA was extracted following the Trizol® method according to the manufacturer’s instructions (Gibco BRL Life Technologies, Baltimore, MD). RNA samples were isolated with RNAeasy minelute cleanup-kit including digestion with DNase I column (Qiagen, Hilden, Germany). After reverse transcript reaction from 1 μg of WAT and BAT mRNA, quantitative realtime reverse transcription polymerase chain reaction (RT-qPCR) was performed in a CFX96TM Real-Time PCR Detection System (Bio-Rad, Hercules, CA) and the FAM dye label format for the TaqMan® Gene Expression Assays (Applied Biosystems). Melting curves analysis was performed to ensure that only a single product was amplified. After analyzing several control genes, values obtained from eWAT samples were normalized in relation to glyceraldehyde 3-phosphate dehydrogenase (*Gapdh*) levels, whereas values obtained from BAT samples were normalized in relation to beta-glucuronidase (*GusB*) levels.

### Western blot analysis

We performed western blotting as described previously (Crespillo et al., 2011b) using specific antibodies (supplementary material Table S4). Tissue portions of eWAT (~100 mg) were homogenized on ice to preserve protein levels. Total protein lysates were subjected to SDS-PAGE on 10% (w/v) SDS gels, electrotransferred on nitrocellulose membranes and probed with the following antibodies: anti-PPARα (cat. no. 20R-PR021, Fitzgerald, Acton, MA), anti-UCP1 (cat. no. sc-6528, Santa Cruz Biotechnology, Santa Cruz, CA), anti-p38 MAPK (cat. no. ab7952-1, Abcam, Cambridge, UK), anti-phospho-p38 MAPK (cat. no. ab32557, Abcam), anti-PRDM16 (cat. no. ab106410, Abcam), anti-β-actin (cat. no. A5316, Sigma, St Louis, MO) and anti-γ-adaptin (cat. no. A36129, BD Biosciences, Franklin Lakes, NJ). Values were expressed in relation to β-actin or γ-adaptin depending on the molecular weight.

### Immunofluorescence

We performed immunofluorescence as described previously (Crespillo et al., 2011a) using specific antibodies (supplementary material Table S4). Paraffined tissue microarray blocks (Manual Tissue Arrayer MTA-1, Beecher Instruments, Inc., Sun Prairie, WI) of eWAT were analyzed for the presence and quantification of PPARα receptor and UCP1 by immunofluorescence and densitometry. The primary antibodies were: anti-PPARα (diluted 1:100, cat. no. 20R-PR021, Fitzgerald) and anti-UCP1 (diluted 1:100, cat. no. sc-6528, Santa Cruz).

Digital high-resolution microphotographs of the eWAT were taken under the same optimized conditions of high-sensitivity fluorescence detection by an Olympus BX41 microscope equipped with an Olympus DP70 digital camera (Olympus Europa GmbH, Hamburg, Germany) and an Olympus X-Cite fluorescence system (X-Cite series 120Q, Olympus). Quantifications of immunofluorescence were carried out by measuring densitometry of the images obtained from replicates and samples: two replicates per sample, eight samples per group and four groups, using the analysis software ImageJ 1.38x (National Institutes of Health, Bethesda, MA).

### Statistical analysis

All data are represented as mean±s.e.m. (standard error of the mean) of at least eight determinations per experimental group (*n*=8). Kolmogorov-Smirnov normality tests indicated that all data followed a Gaussian distribution (*P*>0.1), so we selected a parametric statistical test. Differences between the treatments were analyzed by ANOVAs and post-hoc two-tailed Bonferroni test. *P*<0.05 was considered significant.

## Supplementary Material

Supplementary Material

## References

[b1-0070129] Alonso M. Serrano A. Vida M. Crespillo A. Hernandez-Folgado L. Jagerovic N. Goya P. Reyes-Cabello C. Perez-Valero V. Decara J. (2012). Antiobesity efficacy of LH-21, a cannabinoid CB(1) receptor antagonist with poor brain penetration, in diet-induced obese rats. Br. J. Pharmacol. 165, 2274–2291 21951309 10.1111/j.1476-5381.2011.01698.x PMC3413862

[b2-0070129] Arch J. R. Wilson S. (1996). Prospects for beta 3-adrenoceptor agonists in the treatment of obesity and diabetes. Int. J. Obes. Relat. Metab. Disord. 20, 191–199 8653138

[b3-0070129] Bachman E. S. Dhillon H. Zhang C. Y. Cinti S. Bianco A. C. Kobilka B. K. Lowell B. B. (2002). betaAR signaling required for diet-induced thermogenesis and obesity resistance. Science 297, 843–845 12161655 10.1126/science.1073160

[b4-0070129] Badman M. K. Pissios P. Kennedy A. R. Koukos G. Flier J. S. Maratos-Flier E. (2007). Hepatic fibroblast growth factor 21 is regulated by PPARalpha and is a key mediator of hepatic lipid metabolism in ketotic states. Cell Metab. 5, 426–437 17550778 10.1016/j.cmet.2007.05.002

[b5-0070129] Barbera M. J. Schluter A. Pedraza N. Iglesias R. Villarroya F. Giralt M. (2001). Peroxisome proliferator-activated receptor alpha activates transcription of the brown fat uncoupling protein-1 gene. A link between regulation of the thermogenic and lipid oxidation pathways in the brown fat cell. J. Biol. Chem. 276, 1486–1493 11050084 10.1074/jbc.M006246200

[b6-0070129] Cannon B. Nedergaard J. (2004). Brown adipose tissue: function and physiological significance. Physiol. Rev. 84, 277–359 14715917 10.1152/physrev.00015.2003

[b7-0070129] Castillo-Meléndez M. McKinley M. J. Summers R. J. (2000). Intracerebroventricular administration of the beta(3)-adrenoceptor agonist CL 316243 causes Fos immunoreactivity in discrete regions of rat hypothalamus. Neurosci. Lett. 290, 161–164 10963888 10.1016/s0304-3940(00)01359-8

[b8-0070129] Chartoumpekis D. V. Habeos I. G. Ziros P. G. Psyrogiannis A. I. Kyriazopoulou V. E. Papavassiliou A. G. (2011). Brown adipose tissue responds to cold and adrenergic stimulation by induction of FGF21. Mol. Med. 17, 736–740 21373720 10.2119/molmed.2011.00075 PMC3146611

[b9-0070129] Cinti S. (2009). Reversible physiological transdifferentiation in the adipose organ. Proc. Nutr. Soc. 68, 340–349 19698198 10.1017/S0029665109990140

[b10-0070129] Coskun T. Bina H. A. Schneider M. A. Dunbar J. D. Hu C. C. Chen Y. Moller D. E. Kharitonenkov A. (2008). Fibroblast growth factor 21 corrects obesity in mice. Endocrinology 149, 6018–6027 18687777 10.1210/en.2008-0816

[b11-0070129] Cousin B. Cinti S. Morroni M. Raimbault S. Ricquier D. Pénicaud L. Casteilla L. (1992). Occurrence of brown adipocytes in rat white adipose tissue: molecular and morphological characterization. J. Cell Sci. 103, 931–942 1362571 10.1242/jcs.103.4.931

[b12-0070129] Crespillo A. Alonso M. Vida M. Pavón F. J. Serrano A. Rivera P. Romero-Zerbo Y. Fernández-Llebrez P. Martínez A. Pérez-Valero V. (2011a). Reduction of body weight, liver steatosis and expression of stearoyl-CoA desaturase 1 by the isoflavone daidzein in diet-induced obesity. Br. J. Pharmacol. 164, 1899–1915 21557739 10.1111/j.1476-5381.2011.01477.x PMC3246714

[b13-0070129] Crespillo A. Suárez J. Bermúdez-Silva F. J. Rivera P. Vida M. Alonso M. Palomino A. Lucena M. A. Serrano A. Pérez-Martín M. (2011b). Expression of the cannabinoid system in muscle: effects of a high-fat diet and CB1 receptor blockade. Biochem. J. 433, 175–185 20955176 10.1042/BJ20100751

[b14-0070129] Dallner O. S. Chernogubova E. Brolinson K. A. Bengtsson T. (2006). Beta3-adrenergic receptors stimulate glucose uptake in brown adipocytes by two mechanisms independently of glucose transporter 4 translocation. Endocrinology 147, 5730–5739 16959848 10.1210/en.2006-0242

[b15-0070129] Decara J. M. Romero-Cuevas M. Rivera P. Macias-González M. Vida M. Pavón F. J. Serrano A. Cano C. Fresno N. Pérez-Fernández R. (2012). Elaidyl-sulfamide, an oleoylethanolamide-modelled PPARα agonist, reduces body weight gain and plasma cholesterol in rats. Dis. Model. Mech. 5, 660–670 22736460 10.1242/dmm.009233 PMC3424463

[b16-0070129] Feldmann H. M. Golozoubova V. Cannon B. Nedergaard J. (2009). UCP1 ablation induces obesity and abolishes diet-induced thermogenesis in mice exempt from thermal stress by living at thermoneutrality. Cell Metab. 9, 203–209 19187776 10.1016/j.cmet.2008.12.014

[b17-0070129] Fu J. Gaetani S. Oveisi F. Lo Verme J. Serrano A. Rodríguez De Fonseca F. Rosengarth A. Luecke H. Di Giacomo B. Tarzia G. (2003). Oleylethanolamide regulates feeding and body weight through activation of the nuclear receptor PPAR-alpha. Nature 425, 90–93 12955147 10.1038/nature01921

[b18-0070129] Granneman J. G. Li P. Zhu Z. Lu Y. (2005). Metabolic and cellular plasticity in white adipose tissue I: effects of beta3-adrenergic receptor activation. Am. J. Physiol. 289, E608–E616 10.1152/ajpendo.00009.2005 15941787

[b19-0070129] Grujic D. Susulic V. S. Harper M. E. Himms-Hagen J. Cunningham B. A. Corkey B. E. Lowell B. B. (1997). Beta3-adrenergic receptors on white and brown adipocytes mediate beta3-selective agonist-induced effects on energy expenditure, insulin secretion, and food intake. A study using transgenic and gene knockout mice. J. Biol. Chem. 272, 17686–17693 9211919 10.1074/jbc.272.28.17686

[b20-0070129] Gutiérrez-Juárez R. Pocai A. Mulas C. Ono H. Bhanot S. Monia B. P. Rossetti L. (2006). Critical role of stearoyl-CoA desaturase-1 (SCD1) in the onset of diet-induced hepatic insulin resistance. J. Clin. Invest. 116, 1686–1695 16741579 10.1172/JCI26991 PMC1464900

[b21-0070129] Guzmán M. Lo Verme J. Fu J. Oveisi F. Blázquez C. Piomelli D. (2004). Oleoylethanolamide stimulates lipolysis by activating the nuclear receptor peroxisome proliferator-activated receptor alpha (PPAR-alpha). J. Biol. Chem. 279, 27849–27854 15123613 10.1074/jbc.M404087200

[b22-0070129] Himms-Hagen J. Melnyk A. Zingaretti M. C. Ceresi E. Barbatelli G. Cinti S. (2000). Multilocular fat cells in WAT of CL-316243-treated rats derive directly from white adipocytes. Am. J. Physiol. 279, C670–C681 10.1152/ajpcell.2000.279.3.C670 10942717

[b23-0070129] Hondares E. Iglesias R. Giralt A. Gonzalez F. J. Giralt M. Mampel T. Villarroya F. (2011a). Thermogenic activation induces FGF21 expression and release in brown adipose tissue. J. Biol. Chem. 286, 12983–12990 21317437 10.1074/jbc.M110.215889 PMC3075644

[b24-0070129] Hondares E. Rosell M. Díaz-Delfín J. Olmos Y. Monsalve M. Iglesias R. Villarroya F. Giralt M. (2011b). Peroxisome proliferator-activated receptor α (PPARα) induces PPARγ coactivator 1α (PGC-1α) gene expression and contributes to thermogenic activation of brown fat: involvement of PRDM16. J. Biol. Chem. 286, 43112–43122 22033933 10.1074/jbc.M111.252775 PMC3234861

[b25-0070129] Imbernon M. Beiroa D. Vázquez M. J. Morgan D. A. Veyrat-Durebex C. Porteiro B. Díaz-Arteaga A. Senra A. Busquets S. Velásquez D. A. (2013). Central melanin-concentrating hormone influences liver and adipose metabolism via specific hypothalamic nuclei and efferent autonomic/JNK1 pathways. Gastroenterology 144, 636–649, e6 23142626 10.1053/j.gastro.2012.10.051 PMC3663042

[b26-0070129] Inokuma K. Okamatsu-Ogura Y. Omachi A. Matsushita Y. Kimura K. Yamashita H. Saito M. (2006). Indispensable role of mitochondrial UCP1 for antiobesity effect of beta3-adrenergic stimulation. Am. J. Physiol. 290, E1014–E1021 10.1152/ajpendo.00105.2005 16368788

[b27-0070129] Ishibashi J. Seale P. (2010). Medicine. Beige can be slimming. Science 328, 1113–1114 20448151 10.1126/science.1190816 PMC2907667

[b28-0070129] Jiang G. Li Z. Liu F. Ellsworth K. Dallas-Yang Q. Wu M. Ronan J. Esau C. Murphy C. Szalkowski D. (2005). Prevention of obesity in mice by antisense oligonucleotide inhibitors of stearoyl-CoA desaturase-1. J. Clin. Invest. 115, 1030–1038 15761499 10.1172/JCI23962 PMC1062893

[b29-0070129] Kharitonenkov A. Shiyanova T. L. Koester A. Ford A. M. Micanovic R. Galbreath E. J. Sandusky G. E. Hammond L. J. Moyers J. S. Owens R. A. (2005). FGF-21 as a novel metabolic regulator. J. Clin. Invest. 115, 1627–1635 15902306 10.1172/JCI23606 PMC1088017

[b30-0070129] Klein J. Fasshauer M. Benito M. Kahn C. R. (2000). Insulin and the beta3-adrenoceptor differentially regulate uncoupling protein-1 expression. Mol. Endocrinol. 14, 764–773 10847579 10.1210/mend.14.6.0477

[b31-0070129] Kumar M. V. Moore R. L. Scarpace P. J. (1999). Beta3-adrenergic regulation of leptin, food intake, and adiposity is impaired with age. Pflugers Arch. 438, 681–688 10555566

[b32-0070129] Lee Y. H. Petkova A. P. Mottillo E. P. Granneman J. G. (2012). In vivo identification of bipotential adipocyte progenitors recruited by β3-adrenoceptor activation and high-fat feeding. Cell Metab. 15, 480–491 22482730 10.1016/j.cmet.2012.03.009 PMC3322390

[b33-0070129] Li P. Zhu Z. Lu Y. Granneman J. G. (2005). Metabolic and cellular plasticity in white adipose tissue II: role of peroxisome proliferator-activated receptor-alpha. Am. J. Physiol. 289, E617–E626 10.1152/ajpendo.00010.2005 15941786

[b34-0070129] Lowell B. B. Spiegelman B. M. (2000). Towards a molecular understanding of adaptive thermogenesis. Nature 404, 652–660 10766252 10.1038/35007527

[b35-0070129] Macotela Y. Emanuelli B. Mori M. A. Gesta S. Schulz T. J. Tseng Y. H. Kahn C. R. (2012). Intrinsic differences in adipocyte precursor cells from different white fat depots. Diabetes 61, 1691–1699 22596050 10.2337/db11-1753 PMC3379665

[b36-0070129] Mandard S. Müller M. Kersten S. (2004). Peroxisome proliferator-activated receptor alpha target genes. Cell. Mol. Life Sci. 61, 393–416 14999402 10.1007/s00018-003-3216-3 PMC11138883

[b37-0070129] Martínez de Ubago M. García-Oya I. Pérez-Pérez A. Canfrán-Duque A. Quintana-Portillo R. Rodríguez de Fonseca F. González-Yanes C. Sánchez-Margalet V. (2009). Oleoylethanolamide, a natural ligand for PPAR-alpha, inhibits insulin receptor signalling in HTC rat hepatoma cells. Biochim. Biophys. Acta 1791, 740–745 19345745 10.1016/j.bbalip.2009.03.014

[b38-0070129] Murano I. Barbatelli G. Giordano A. Cinti S. (2009). Noradrenergic parenchymal nerve fiber branching after cold acclimatisation correlates with brown adipocyte density in mouse adipose organ. J. Anat. 214, 171–178 19018882 10.1111/j.1469-7580.2008.01001.x PMC2667925

[b39-0070129] Oberkofler H. Dallinger G. Liu Y. M. Hell E. Krempler F. Patsch W. (1997). Uncoupling protein gene: quantification of expression levels in adipose tissues of obese and non-obese humans. J. Lipid Res. 38, 2125–2133 9374134

[b40-0070129] Petrovic N. Walden T. B. Shabalina I. G. Timmons J. A. Cannon B. Nedergaard J. (2010). Chronic peroxisome proliferator-activated receptor gamma (PPARgamma) activation of epididymally derived white adipocyte cultures reveals a population of thermogenically competent, UCP1-containing adipocytes molecularly distinct from classic brown adipocytes. J. Biol. Chem. 285, 7153–7164 20028987 10.1074/jbc.M109.053942 PMC2844165

[b41-0070129] Ricquier D. Bouillaud F. (2000). The uncoupling protein homologues: UCP1, UCP2, UCP3, StUCP and AtUCP. Biochem. J. 345, 161–179 10620491 PMC1220743

[b42-0070129] Rodríguez de Fonseca F. Navarro M. Gómez R. Escuredo L. Nava F. Fu J. Murillo-Rodríguez E. Giuffrida A. LoVerme J. Gaetani S. (2001). An anorexic lipid mediator regulated by feeding. Nature 414, 209–212 11700558 10.1038/35102582

[b43-0070129] Schwartz G. J. Fu J. Astarita G. Li X. Gaetani S. Campolongo P. Cuomo V. Piomelli D. (2008). The lipid messenger OEA links dietary fat intake to satiety. Cell Metab. 8, 281–288 18840358 10.1016/j.cmet.2008.08.005 PMC2572640

[b44-0070129] Seale P. Kajimura S. Yang W. Chin S. Rohas L. M. Uldry M. Tavernier G. Langin D. Spiegelman B. M. (2007). Transcriptional control of brown fat determination by PRDM16. Cell Metab. 6, 38–54 17618855 10.1016/j.cmet.2007.06.001 PMC2564846

[b45-0070129] Seale P. Bjork B. Yang W. Kajimura S. Chin S. Kuang S. Scimè A. Devarakonda S. Conroe H. M. Erdjument-Bromage H. (2008). PRDM16 controls a brown fat/skeletal muscle switch. Nature 454, 961–967 18719582 10.1038/nature07182 PMC2583329

[b46-0070129] Seale P. Conroe H. M. Estall J. Kajimura S. Frontini A. Ishibashi J. Cohen P. Cinti S. Spiegelman B. M. (2011). Prdm16 determines the thermogenic program of subcutaneous white adipose tissue in mice. J. Clin. Invest. 121, 96–105 21123942 10.1172/JCI44271 PMC3007155

[b47-0070129] Skurk T. Alberti-Huber C. Herder C. Hauner H. (2007). Relationship between adipocyte size and adipokine expression and secretion. J. Clin. Endocrinol. Metab. 92, 1023–1033 17164304 10.1210/jc.2006-1055

[b48-0070129] Tiraby C. Tavernier G. Lefort C. Larrouy D. Bouillaud F. Ricquier D. Langin D. (2003). Acquirement of brown fat cell features by human white adipocytes. J. Biol. Chem. 278, 33370–33376 12807871 10.1074/jbc.M305235200

[b49-0070129] Tsujii S. Bray G. A. (1992). Food intake of lean and obese Zucker rats following ventricular infusions of adrenergic agonists. Brain Res. 587, 226–232 1356064 10.1016/0006-8993(92)91001-u

[b50-0070129] Vegiopoulos A. Müller-Decker K. Strzoda D. Schmitt I. Chichelnitskiy E. Ostertag A. Berriel Diaz M. Rozman J. Hrabe de Angelis M. Nüsing R. M. (2010). Cyclooxygenase-2 controls energy homeostasis in mice by de novo recruitment of brown adipocytes. Science 328, 1158–1161 20448152 10.1126/science.1186034

[b51-0070129] Villarroya F. Iglesias R. Giralt M. (2007). PPARs in the control of uncoupling proteins gene expression. PPAR Res. 2007, 74364 17389766 10.1155/2007/74364 PMC1779581

[b52-0070129] Virtanen K. A. Lidell M. E. Orava J. Heglind M. Westergren R. Niemi T. Taittonen M. Laine J. Savisto N. J. Enerbäck S. (2009). Functional brown adipose tissue in healthy adults. N. Engl. J. Med. 360, 1518–1525 19357407 10.1056/NEJMoa0808949

[b53-0070129] White C. L. Ishihara Y. Dotson T. L. Hughes D. A. Bray G. A. York D. A. (2004). Effect of a beta-3 agonist on food intake in two strains of rats that differ in susceptibility to obesity. Physiol. Behav. 82, 489–496 15276814 10.1016/j.physbeh.2004.04.059

[b54-0070129] Zhang Y. Matheny M. Zolotukhin S. Tumer N. Scarpace P. J. (2002). Regulation of adiponectin and leptin gene expression in white and brown adipose tissues: influence of beta3-adrenergic agonists, retinoic acid, leptin and fasting. Biochim. Biophys. Acta 1584, 115–122 12385894 10.1016/s1388-1981(02)00298-6

