# Supplementary material for: Oleoylethanolamide enhances β-adrenergic-mediated thermogenesis and white-to-brown adipocyte phenotype in epididymal white adipose tissue in rat
Source: Dis Model Mech. 2013 Oct 23;7(1):129–41. doi: 10.1242/dmm.013110 (PMC3882055; doi:10.1242/dmm.013110)
Supplement: Supplementary Material [file supp_7_1_129__index.html]

Oleoylethanolamide enhances β-adrenergic-mediated thermogenesis and white-to-brown adipocyte phenotype in epididymal white adipose tissue in rat — Supplementary Material 

# Oleoylethanolamide enhances β-adrenergic-mediated thermogenesis and white-to-brown adipocyte phenotype in epididymal white adipose tissue in rat

## DMM013110 Supplementary Material

**Files in this Data Supplement:**

- **Supplementary Material PDF**
