# Supplementary material for: Oleoylethanolamide enhances β-adrenergic-mediated thermogenesis and white-to-brown adipocyte phenotype in epididymal white adipose tissue in rat
Source: Dis Model Mech. 2013 Oct 23;7(1):129–41. doi: 10.1242/dmm.013110 (PMC3882055; doi:10.1242/dmm.013110)
Supplement: Supplementary Material [file supp_013110_DMM013110.pdf]

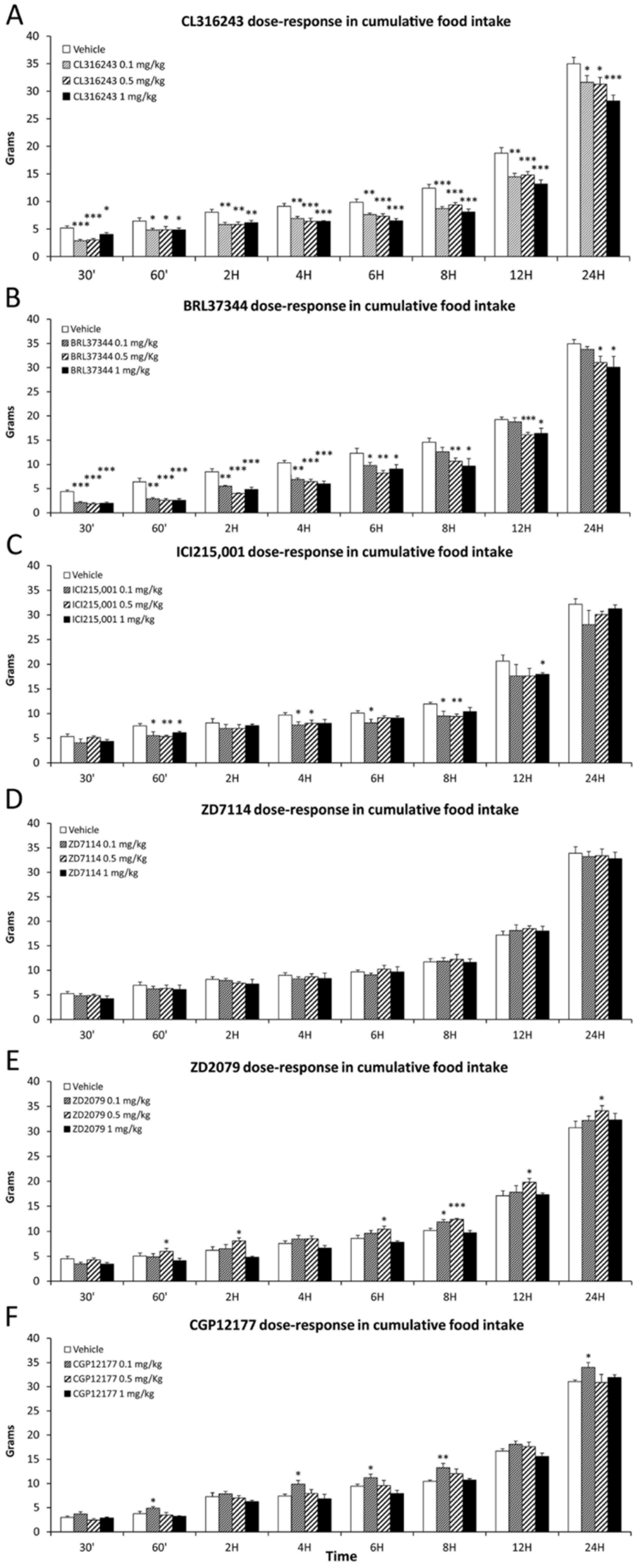

Fig. S1. Dose-response effects of acute administration of the β3 adrenoceptor agonists CL316243, BRL37344, ICI215,001, ZD7114, ZD2079 and CGP12177 at doses of 0.1, 0.5 and 1 mg/kg on cumulative food intake over 24 hours in rats food-deprived for 24 hours. Histograms represent the mean±s.e.m. (n=8). *P<0.05, **P<0.01, ***P<0.001 versus vehicle-treated rats.

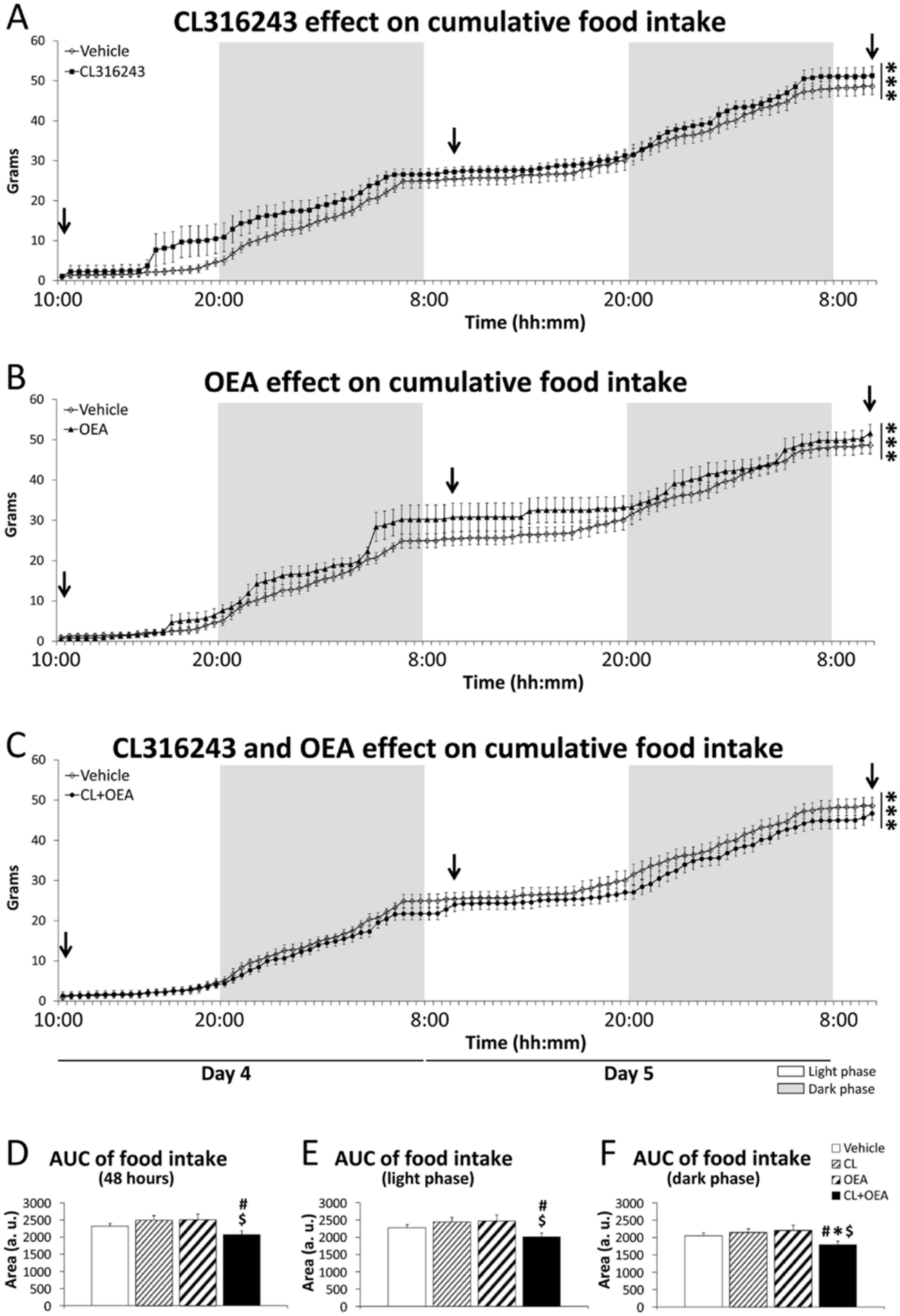

Fig. S2. Effects of repeated administration of CL316243 (1 mg/kg) and/or OEA (5 mg/kg) on cumulative food intake (A-C) for 48 hours after 4 days of treatment. Area under the curve (AUC) of food intake (D-F) for 48 hours and during light and dark phase. Histograms represent the mean±s.e.m. (*n*=8). **P*<0.05, ****P*<0.001 versus vehicle-treated rats; #*P*<0.05 versus CL316243-treated rats; $*P*<0.05 versus OEA-treated rats.

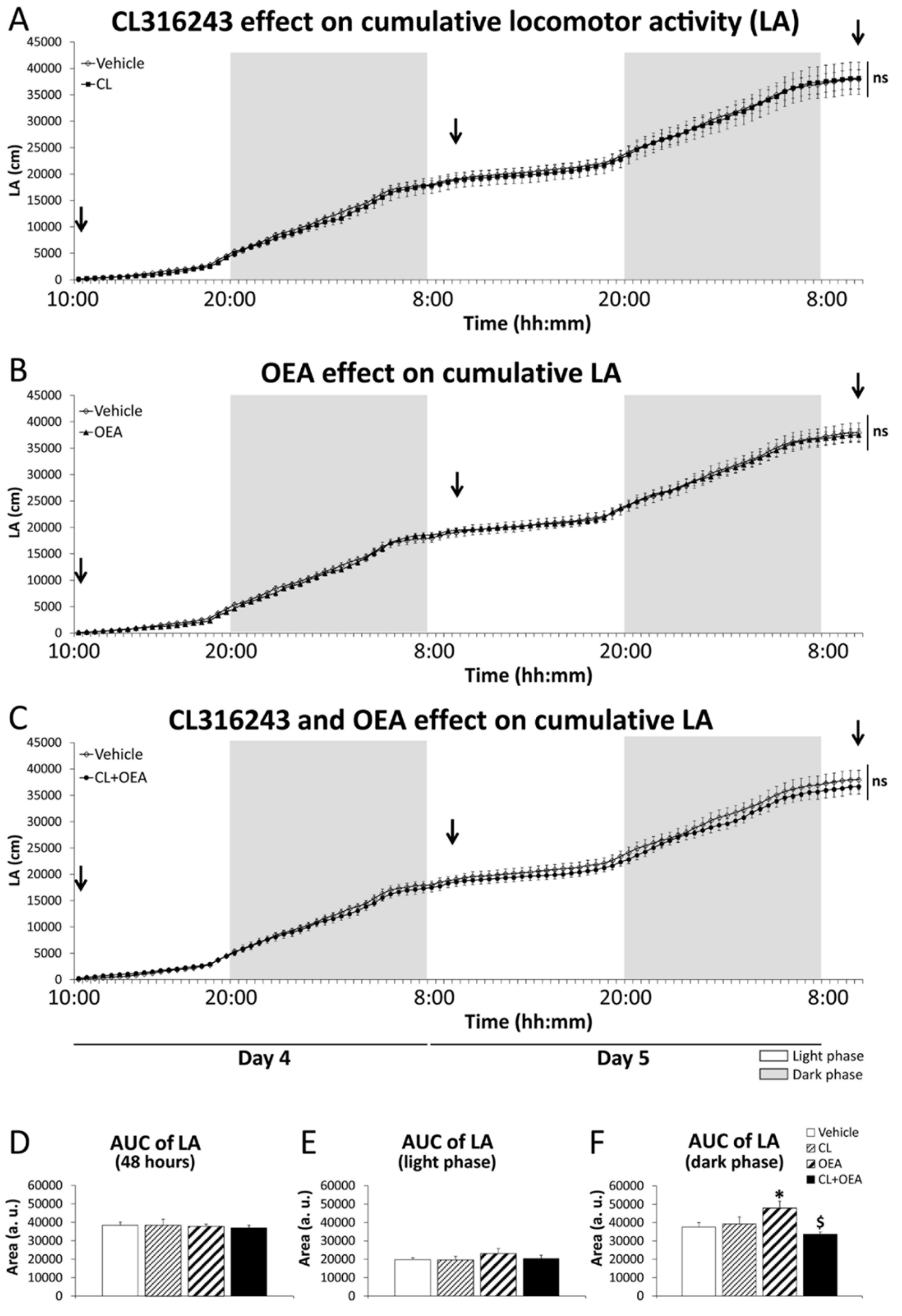

Fig. S3. Effects of repeated administration of CL316243 (1 mg/kg) and/or OEA (5 mg/kg) on cumulative locomotor activity (LA) (A-C) for 48 hours after 4 days of treatment. Area under the curve (AUC) of LA (D-F) for 48 hours and during light and dark phase. Histograms represent the mean±s.e.m. (*n*=8). *P<0.05 versus vehicle-treated rats; $^{\$}P$<0.05 versus OEA-treated rats.

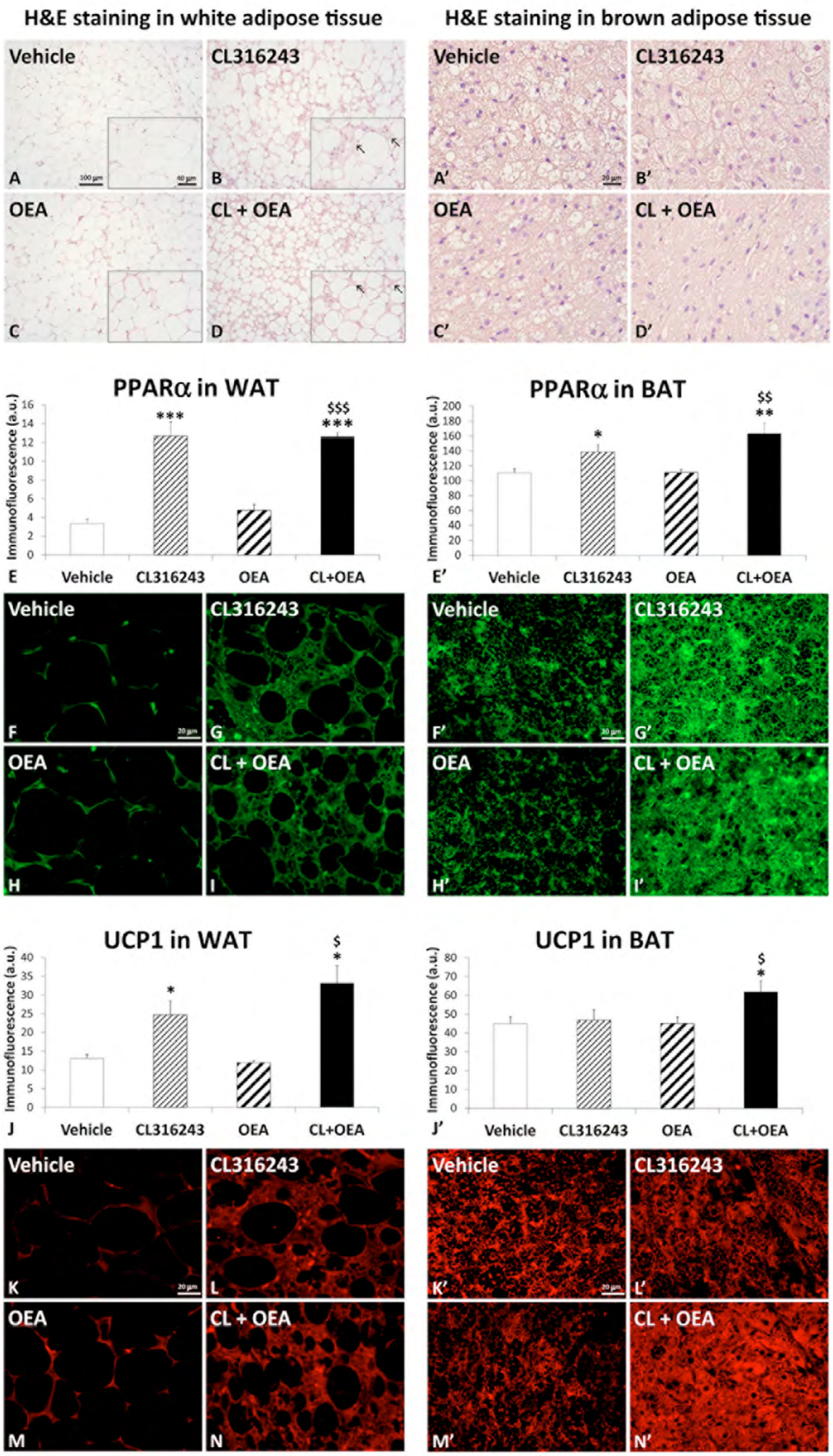

Fig. S4. Effects of repeated administration of CL316243 and/or OEA on the morphology of the white and brown adipocytes and their immunofluorescent levels of PPARα and UCP1 after 6 days of treatment. Arrows point to multilocular adipocytes. (A,A′-D,D′) Hematoxylin and eosin (H&E) staining in WAT and BAT. (E,E′-I,I′) Quantification of PPARα immunofluorescence and representative images of WAT and BAT. (J,J′-N,N′) Quantification of UCP1 immunofluorescence and representative images of WAT and BAT. Histograms represent the mean±s.e.m. (*n*=8). **P*<0.05, ***P*<0.01, ****P*<0.001 versus vehicle-treated rats; $^{\$}P<0.05$, $^{\$\$}P<0.01$, $^{\$\$\$}P<0.001$ versus OEA-treated rats.

Table S1. Effect of CL316243 and OEA on total liver fat.[1]

	**Vehicle**	**CL316243**	**OEA**	**CL+OEA**
**Total fat (%)**	3.63±0.05	3.57±0.04	3.69±0.08	3.73±0.09

[1]Percentage of total liver fat in rats treated with vehicle, CL316243 (1 mg/kg), OEA (5 mg/kg) and the combination of CL316243 and OEA. Values represent the mean ± SEM (*n*=8).

Table S2. Primer references for TaqMan® Gene Expression Assays (Applied Biosystems).[1]

Genes	Assay ID	Amplicon length
Acox1	Rn01460628_m1	63
Cox4i1	Rn00665001_g1	72
Cox4i2	Rn00585003_m1	59
Cpt1b	Rn00682395_m1	83
Fasn	Rn00569117_m1	74
Fgf21	Rn04219642_g1	61
Gapdh	Rn01775763_g1	175
GusB	Rn00566655_m1	63
Hmgcr	Rn00565598_m1	71
Insig1	Rn00574380_m1	68
Insig2	Rn00710111_m1	89
Pparα	Rn00566193_m1	98
Scd1	Rn00594894_g1	86
Srebf1	Rn01495769_m1	79
Srebf2	Rn01502638_m1	61
Ucp1	Rn00562126_m1	69

[1]*Acox1*, acyl-Coenzyme A oxidase 1, palmitoyl; Cox4i1, cytochrome c oxidase subunit IV isoform 1; Cox4i2, cytochrome c oxidase subunit IV isoform 2; *Cpt1b*, carnitine palmitoyltransferase 1b, muscle; *Fasn*, fatty acid synthase; *Fgf21*, fibroblast growth factor 21; *Gapdh*, glyceraldehyde-3-phosphate dehydrogenase; *GusB*, beta-glucuronidase; *Hmgcr,* 3-hydroxy-3-methylglutaryl-CoA reductase; *Insig1/2*, insulin induced gene 1/2; *Pparα*, peroxisome proliferator activated receptor alpha; *Scd1*, stearoyl-Coenzyme A desaturase 1; *Srebf1/2*, sterol regulatory element binding transcription factor 1/2; *Ucp1*, uncoupling protein 1 (mitochondrial, proton carrier).

Table S3. Primers sequences for qPCR designed based on NCBI database sequences of rat reference predicted mRNA, checked for specificity with BLAST software from NCBI website (http://blast.ncbi.nlm.nih.gov/Blast.cgi) and synthesized by Eurofins (Ebersberg, Germany).[1]

Genes	GenBank code	Oligonucleotide primers (forward, reverse) and probe	Amplicon length
Prdm16	XM_002726622.1	FW: 5´ CCACACAGAAGAGCGTGAGTACAA<br>RV: 5´ TGTGAACACCTTGACGCAGTTT<br>PB: 5´ TCTCACGACAGTGGCAAGCGCTTC	92

[1]*Prdm16*, PR domain containing 16-like.

Table S4. Antibodies, molecular weights obtained and dilutions used for Western blotting (WB) and/or immunofluorescence (IF).[1]

Antibody	Molecular weight (kD)	Dilution	Host
**PPARα**	55	WB: 1:1000<br>IF: 1:100	Rabbit
**UCP1**	33	WB: 1:1000<br>IF: 1:100	Goat
**PRDM16**	140	WB: 1:500	Rabbit
**p38 MAPK**	38	WB: 1:1000	Rabbit
**p38 MAPK-P**	38	WB: 1:1000	Rabbit
**β-actin**	45	WB: 1:2000	mouse
**γ-adaptin**	102	WB: 1:1000	mouse

[1]ACC(-P), acetyl-CoA carboxylase (-phosporylated); AMPKα(-P), 5' adenosine monophosphate-activated protein kinase (-phosporylated); FAS, fatty acid synthase; p38 MAPK(-P), p38 mitogen-activated protein kinase (-phosporylated); PPARα, peroxisome proliferator activated receptor alpha; PRDM16, PR domain containing 16 like; UCP1, uncoupling protein 1 (mitochondrial, proton carrier).
